# Supplementary material for: Perceptions of emergency care using a seizure care pathway for patients presenting to emergency departments in the North West of England following a seizure: a qualitative study
Source: BMJ Open. 2018 Sep 28;8(9):e021246. doi: 10.1136/bmjopen-2017-021246 (PMC6169770; doi:10.1136/bmjopen-2017-021246)
Supplement: Author's manuscript [file bmjopen-2017-021246.draft_revisions.pdf]

BMJ Open is committed to open peer review. As part of this commitment we make the peer review history of every article we publish publicly available.

When an article is published we post the peer reviewers' comments and the authors' responses online. We also post the versions of the paper that were used during peer review. These are the versions that the peer review comments apply to.

The versions of the paper that follow are the versions that were submitted during the peer review process. They are not the versions of record or the final published versions. They should not be cited or distributed as the published version of this manuscript.

BMJ Open is an open access journal and the full, final, typeset and author-corrected version of record of the manuscript is available on our site with no access controls, subscription charges or pay-per-view fees (http://bmjopen.bmj.com).

If you have any questions on BMJ Open's open peer review process please email info.bmjopen@bmj.com

BMJ Open

Qualitative process evaluation of a seizure care pathway for patients presenting to ED following a seizure.

Journal:	*BMJ Open*
Manuscript ID	bmjopen-2017-021246
Article Type:	Research
Date Submitted by the Author:	19-Dec-2017

Complete List of Authors:	Male, Leanne; University of Liverpool, Department of Neuroscience<br>Noble, Adam; University of Liverpool, Department of Psychological Sciences<br>Snape, Darlene; University of Liverpool, Department of Psychological Sciences<br>Dixon, Peter; University of Liverpool, Department of Biostatistics<br>Marson, Tony; University of Liverpool, Institute of Translational Medicine; The Walton Centre NHS Foundation Trust

Keywords:	ACCIDENT & EMERGENCY MEDICINE, Organisation of health services < HEALTH SERVICES ADMINISTRATION & MANAGEMENT, Quality in health care < HEALTH SERVICES ADMINISTRATION & MANAGEMENT, Epilepsy < NEUROLOGY, QUALITATIVE RESEARCH

SCHOLARONE™
Manuscripts

V8 14/12/2017

Qualitative process evaluation of a seizure care pathway for patients presenting to ED following a seizure

Authors

Leanne Male *, Dr. Adam Noble *, Dr. Darlene Snape*, Dr. Peter Dixon*, Prof. Tony Marson *,¶

* University of Liverpool, UK ¶ The Walton Centre NHS Foundation Trust, Liverpool, UK

Corresponding Author

Address: Room 2.29, Clinical Sciences Centre, Aintree University Hospital, Lower Lane, Fazakerley, L9 7LJ. **Email:** hllmale@student.liv.ac.uk

Co-Authors

Dr. Adam Noble, University of Liverpool, Department of Psychological Sciences, Liverpool, UK

Dr. Darlene Snape, University of Liverpool, Department of Psychological Sciences, Liverpool, UK

Dr. Peter Dixon, University of Liverpool, Department of Biostatistics, Liverpool, UK

Prof. Tony Marson, University of Liverpool, Institute of Translational Medicine, Liverpool, UK

The Walton Centre NHS Foundation Trust, Liverpool, UK.

Word Count- 6009

Keywords

Emergency medical services, seizures, epilepsy, qualitative research, process evaluation

For peer review only

For peer review only - http://bmjopen.bmj.com/site/about/guidelines.xhtml

Qualitative process evaluation of a seizure care pathway for patients presenting to ED following a seizure

Abstract

Objectives To establish the appropriateness and effectiveness of the seizure care pathway by exploring whether patients valued the intervention, viewed it as being particularly helpful or not, and why, and whether all patient groups received the same treatment and care.

Design Qualitative descriptive study, using semi-structured, in-depth interviews and thematic template analysis, theoretically informed by critical realism.

Setting In North-West England, a seizure care pathway has been developed in collaboration with a specialist neurology hospital, to support clinical management of seizure patients on initial presentation to the emergency department (ED), as well as rapid access to follow-up services on discharge, with an aim to improve patient outcomes and experience. Three National Health Service EDs and a specialist neurology hospital were the setting for recruiting participants to this study.

Participants 27 participants following their experience of an ED attendance and outpatient follow-up appointment after a seizure.

Results Five main themes emerged from the data; decision to seek care, responsiveness of services, waiting and efficiency, information and support, and care continuity. Two integrative themes spanned the whole study; lived experience and communication. This paper reports on two of the main themes; care continuity and waiting and efficiency.

Conclusion Implementation of a care pathway is a complex intervention, requiring long-term follow-up to assess its integration into practice and effectiveness in service improvement. The seizure care pathway has potential to enhance the care of seizure patients in the ED and at follow-up by improving continuation and management of care. The study demonstrates good aspects of the seizure care pathway as observed by patients, but also recognises shortcomings within current service provision and questions what the NHS should and should not be delivering. Our study suggests various ways in which the pathway could potentially be improved at service-level to drive patient experience.

For peer review only - http://bmjopen.bmj.com/site/about/guidelines.xhtml

Strengths and limitations of this study

- This is the first in-depth study exploring patient experience of a seizure care pathway in the UK.
- Patients were recruited after presenting at one of three different NHS trusts and so it is likely that the issues reported do not occur in isolation.
- The study allows the voices of both patients with known epilepsy and those experiencing their first seizure to be heard, as well as their carers/relatives, allowing for data to be collected from multiple viewpoints.
- The study did not capture the perspectives of staff regarding the delivery of the care pathway in practice, which may give further insight into the appropriateness and effectiveness of its use.
- It remains to be established whether addressing the issues raised by participants about their experience of the pathway would ultimately improve the quality of care delivered and overall patient experience.

Introduction

The NHS, like other health systems around the world, is operating within a context of rising demand, slow growth in funding and increasing operating costs. As a consequence, NHS providers ended 2015/16 with a record funding deficit of £2.45 billion [1]. The Five Year Forward View challenges the NHS to make unprecedented savings [2]. One way to achieve this is to reduce avoidable use of expensive emergency hospital services.

In the UK, seizures account for 3.3% of all calls to the emergency ambulance service [3], ~60,000 seizure-related ED attendances and ~40,000 hospital admissions each year, at a cost to the NHS in 2015/16 of ~£70 million [4-6]. Emergency care for seizures can be appropriate, important, and even life-saving. Despite this, evidence suggests that seizure patients' attendance at the emergency department (ED) is, more often than not, clinically unnecessary [7-9].

Frequently, seizure-related ED visits are though expressions of sub-optimal ambulatory care. In the National Audit of Seizure management in Hospitals (NASH), most of those presenting to the ED with known epilepsy (~65%), had not been seen an epilepsy

For peer review only - http://bmjopen.bmj.com/site/about/guidelines.xhtml

specialist within the past 12 months, but were in need of such support. They were often being treated using sub-optimal, outdated drug treatment regimens and so at risk of avoidable seizures and/ or adverse events [4]. NICE (2016) recommend when seizures are not controlled or treatment fails, the patient should be referred to a specialist for assessment [10]. Evidence suggests such input could help improve patient outcomes [11]. ED visits do not, however, currently lead to most attendees receiving such specialist support [4].

Part of the solution might lie with the introduction of a clinical care pathway within acute hospitals. Care pathways have proven to work in the NHS as a means of directing and co-ordinating patient care across health care providers [12]. Importantly, a recent study in the Republic of Ireland saw the implementation of a pathway specifically for seizures. It was designed to improve clinical management, reduce unnecessary admissions, minimise length of stay following ED attendance and improve follow-up services through a rapid access clinic [13]. Initial evidence suggests the pathway has utility in reducing length of stay for patients and time until a patient is followed-up by outpatient services [13].

Ensuring equitable access to specialist neurological care in the UK has long been challenging. The above evidence suggests instituting a more proactive and better coordinated model of care may hold promise in driving up care quality while at the same time reducing emergency service use for seizures. Within the Cheshire and Mersey region, a seizure care pathway was therefore developed. Seizure services in the area had been identified as a key target for improvement and better co-ordination [4]. The Cheshire and Merseyside Strategic Clinical Network (SCN) co-ordinated the development of the pathway for adults presenting to emergency services with seizures in collaboration with stakeholders (neurologists, general practitioners, patient and public representatives and commissioners).

The Care After Presenting with Seizures (CAPS) study, funded by the National Institute of Health Research (CLAHRC NWC), was set up to evaluate the pathway's effect. CAPS is a non-randomised trial comparing the effect of the pathway to usual care alone in reducing the number of emergency visits made by patients with seizures attending three hospital EDs in the North-West of England linked to a tertiary neurology service; The Walton Centre NHS Foundation Trust.

The pathway is though a 'complex intervention'. Such interventions are *"built up from a number of components, which may act both independently and interdependently"*

For peer review only - http://bmjopen.bmj.com/site/about/guidelines.xhtml

[14]. Medical Research Council (MRC) guidance highlights that a full evaluation of such interventions requires consideration of issues in addition to effectiveness [15]. Such information helps understand why an intervention did or did not work, what its ‘‘active ingredients’’ are and how treatment may need refining. They recommend such questions be addressed by a ‘process evaluation’ [15, 16].

This paper presents the findings of a process evaluation of the seizure care pathway. It specifically aimed to explore: 1) whether the pathway was valued by patients and, if so, why?; (2) whether any aspects of the intervention were perceived by patients as being particularly helpful or unhelpful; and, (3) whether all patients who had access to the pathway perceived similar benefits and limitations to the intervention or whether this was dependant on specific patient characteristics.

Methods

Study Design and Setting

This work is a qualitative study conducted with semi-structured patient interviews. We were interested in a descriptive evaluation of the seizure care pathway based on participants' understanding, perceptions and experiences, both in relation to their ED care following a seizure presentation and the subsequent follow-up service offered.

Participants

Table 1 outlines the inclusion/exclusion criteria for the study. Screening was undertaken by LM, a PhD student with a background in nursing.

Table 1

Participant inclusion/exclusion criteria for study

Inclusion Criteria	Exclusion Criteria
• Aged 16 years or older.<br>• Presented in ED following a suspected seizure, regardless of whether they had a previous clinical diagnosis of epilepsy.	• Provoked seizure (e.g. alcohol withdrawal).<br>• Event considered unlikely to be an unprovoked seizure (e.g. syncope).<br>• Reside outside of Walton Centre's catchment area<br>• Learning disability.<br>• Not fluent in written or spoken English.

For peer review only - http://bmjopen.bmj.com/site/about/guidelines.xhtml

Participants were recruited using purposive sampling, as detailed in the results section, to allow representation of the diverse range of patients attending the ED following a seizure [17, 18].

Data Collection

Following ethical approval from Wales Research Ethics Committee (Study Ref. 15/WA/0207), potential participants were contacted to discuss the study further and, if interested, a time and date for interview was arranged. Participant consent was obtained prior to commencement of the interview. Face-to-face interviews were conducted with the participant and, if requested by the participant, a carer - who had been present at the time of the ED attendance – was also invited to contribute.

The interview topic guide (Table 2) was developed through a review of the relevant literature and iteratively updated throughout the process of data collection, through ongoing analysis, researcher reflections and study team discussions [19].

Table 2
Specific topics introduced within interviews.

Figure 1. Topic guide for interviews
Can you tell me about what happened to make you attend ED?<br>Probe: experience, decision-making, transport to ED.
Tell me what you remember about your experience in the ED.<br>Probe: Care processes, interactions with professionals, involvement in decision-making, information and support provision.
Was the most recent experience any different to past experiences of ED attendances?<br>Probe: similarities/differences, likes/dislikes, changes.
What is 'good care' to you?<br>Probe: expectations, shortfalls.<br>What sort of changes, if any, could be made to improve your care throughout different stages in the ED?
Tell me about your experience of the transition of care between ED and Walton Centre?<br>Probe: communication, timeliness, similarities/differences to previous referrals.
Can you tell me a bit about your experience when you went for your outpatient clinic

For peer review only - http://bmjopen.bmj.com/site/about/guidelines.xhtml

appointment?<br>Probe: usefulness of appointment, benefits of pathway, improvement suggestions, how well do you feel your 'crisis' was dealt with?
Discuss the utility of the current ED Questionnaire (Picker Institute) from patients' opinion[20].

Data analysis

Interviews were analysed thematically using the template method, informed by the work of King [21]. QSR International's NVivo V.10 software [22] was used to facilitate data management and document the analytical process. The analytic template was developed using a combination of *a priori* codes, derived from previous research around patient experience in general, and salient findings from early participant interviews. Using *a priori* codes ensured a focus on key areas potentially relevant to the study [23]. Further codes and themes were identified deductively through analysis of raw data, allowing identification of themes and patterns related to the study aims [24].

The initial template was developed by LM and another researcher (DS). Transcripts were coded separately by LM and DS before comparison was made using the coding template [25]. Transcripts were read and re-read, further amendments were made to the template until coding consensus was reached [26, 27]. Final themes were reviewed by the study team to ensure they addressed the subject matter and accounted for the content within the data.

Findings

Participant characteristics

A total of twenty seven interviews were conducted between March 2016 and May 2017. Twenty five of the interviews were conducted in the participant's own home, with the remaining two interviews, taking place at an alternative location at the request of the participant. Demographics and clinical characteristics of the study sample are outlined in Table 3.

For peer review only - http://bmjopen.bmj.com/site/about/guidelines.xhtml

Table 3

Patient demographics and clinical characteristics, grouped according to diagnosis following neurology review.

	Known Epilepsy (n=13)	First unprovoked Seizure (n= 8)	Not epileptic Seizure (n=6)
**Age (years)**			
-Mean	50	37	32
-Range	24-78	17-64	19-45
**Gender**			
-Male	6 (46.2%)	5 (62.5%)	4 (66.7%)
-Female	7 (53.8%)	3 (37.5%)	2 (33.3%)
**Employment**			
-Full time work	3 (23.1%)	1 (12.5%)	2 (33.3%)
-Part time work	2 (15.4%)	1 (12.5%)	0 (0%)
-Not employed	8 (61.5%)	5 (62.5%)	3 (50%)
-Education	0 (0%)	1 (12.5%)	1 (16.7%)
**IMD Regional Decile**			
-1-3 (most deprived)	9 (69.2%)	4 (50%)	3 (50%)
-4-6	2 (15.4%)	3 (37.5%)	2 (33.3%)
-7-10 (least deprived)	2 (15.4%)	1 (12.5%)	1 (16.7%)

Identified themes

Analysis of the transcripts elicited five key concepts (Figure 1). In addition, two integrative themes: 'Lived Experience' and 'Communication' were found to be common to all themes and permeated participant discussions whatever the foreground; proving important throughout the holistic analytic process.

The present paper focuses on findings from two of the five top level themes; namely, 'waiting and efficiency' and 'continuity of care'. Care continuity, both within the ED and across services, was highlighted as the most important to patients in relation to their overall experience of the care pathway, along with waiting, which was frequently referred to throughout participant interviews. Each theme/sub-theme will be discussed in turn in relation to participants' perception of the pathway as an effective intervention and examine each in relation to its perceived value to the pathway process overall.

For peer review only - http://bmjopen.bmj.com/site/about/guidelines.xhtml

Waiting and efficiency

Time was viewed by most participants as a factor influencing experience. Irrespective of whether the participant perceived their treatment as being fast or slow, time was continually mentioned, highlighting its importance in the process experienced by participants.

Participants' experience of waiting times varied at different points in their journey through the ED. Reference was repeatedly made to '*waiting for triage*', '*waiting for tests*' and '*waiting to see a doctor*'. When participants perceived their wait time as being '*quick*', particularly in comparison to previous experience, they reported positive feedback on the process.

> *'there was no question of she's going to wait or whatever; come right through and I say I saw that doctor…they were absolutely brilliant I couldn't fault them at all.'* (P8, Female, First unprovoked seizure)

Conversely, participants perceived 'long' waiting contributed to poor experience.

> *'if your waiting on one of them beds to see a doctor…it does make you feel a bit on edge…people just get upset sitting out there.'* (P29, Male, Known epilepsy)

Waiting and environmental resources

Participants frequently described the physical environment of the ED as "*busy*". A commonly reported feature was lack of bed space within the department, resulting in participants being left on trollies in the ED corridor for significant periods of time. As one female explained:

> *'they were so backed up that we came in via ambulance and you know the way they have all the trolleys down the corridor backed up, they were that bad that the paramedics had to stay with me for 3 hours in a corridor.'* (P5, Female, Known epilepsy)

Similarly, this scenario had a significant impact on another participant's ED experience, prompting his self-discharge from the service. In this instance, the participant reported

For peer review only - http://bmjopen.bmj.com/site/about/guidelines.xhtml

being in a post-ictal state but felt he no longer particularly required treatment; a decision informed, in part, by the belief he was a burden to the paramedics who had to remain with him during his time on a trolley in the corridor. He explained:

> '*there was the guilt trip I suppose on the last one because I had, this was adding to the anxiety because I was stuck on a trolley with paramedics looking after me and I'm thinking they could be out saving someone else's life. So that added to the pressure if that makes sense. Erm and that's why I said look you can't wait any longer and so I signed a self-discharge form because I felt guilty*.' (P32, Male, Known epilepsy)

Further interviews revealed participants' observations about the environment (often described as "*busy*" and "*noisy*") as well as their views about how it could be improved in a way that was more conducive to supporting those following a seizure. Suggestions for side rooms, dimmed lighting, and a quieter area to enable patients to recover from their seizure in a calm and timely manner were articulated.

> *'…they realised then they had to put me in a dark room to take my bloods and everything and then they said we'll try and put you somewhere quiet… they moved me up to the next waiting area… again there was the television on, flashing lights and we said you know you can't do that. If you put me in a dark room where it is nice and quiet my seizures will stop and calm down but they won't listen*.' (P5, Female, Known epilepsy)

Time for care

Participants not only spoke about time in relation to waiting, but also with reference to time health care professionals (HCPs) spent engaging with them. While the majority of participants reported being satisfied with the time spent with HCPs, the visibility of staff members, and the communication they received, others spoke of negative experiences related to the limited time spent specifically with HCPs.

Participants reported that waiting for a doctor to arrive often induced fear and uncertainty; triggering worrying thoughts about what might be wrong and what would happen next. This feeling was articulated mainly by those participants experiencing a first seizure, for whom the event and ED environment was largely unfamiliar. Overall,

For peer review only - http://bmjopen.bmj.com/site/about/guidelines.xhtml

participants commonly held the view that doctors and nurses were "*in a rush*". Such observations and experiences led to a sense of "*urgency*", felt by participants and a perceived inadequacy in support offered by HCPs.

> *'I don't know how long the doctor was with me or the nurse but it didn't seem very long at all… yes I would have liked to have known more and the amount of time I spend in there, it seemed a very little time I spent with the doctor or the nurse.'* (P12, Male, Known epilepsy)

Conversely, those with known epilepsy were more relaxed about the visibility of HCPs, not least because they expressed being more *"in control*" of the situation. More often than not, these participants reported a more positive experience of visibility of HCPs and were happy with the amount of time spent in consultation with doctors and nurses; even if this time was minimal and contact related mainly to routine basic observations.

> *'we were pretty much left to our own devices really, they checked that she was ok and then off they went. They weren't really regular check-ups because its more just waiting for her to come back round and be lucid and waiting for a doctor to turn up.'* (P6, Female, Known epilepsy, Carer)

In contrast a female with known epilepsy expressed great concern about the lack of contact with HCP's. Her concern led to fears for safety and a sense of heightened insecurity about the level of care received. These fears occasioned a negative perception of the care pathway and general experience within the ED.

> '*there was no medical staff available because they were so busy… I felt in danger because nobody knew what they were doing.'* (P5, Female, Known epilepsy)

Conceptualisation of service provision

Despite acknowledging shortfalls in the service provided, often participants expressed understanding as to why this may be the case. Most commonly, lack of staff and resources in the ED were highlighted as a potential cause of increased waiting times.

For peer review only - http://bmjopen.bmj.com/site/about/guidelines.xhtml

'it was just queue of trollies with people in it. It was 4 and a half/5 hours just sat, lying around waiting… I didn't see anyone I was just lying there.' (P26, Male, First unprovoked seizure)

Participants also noted 'time of day' at which they attended the ED as a causal factor for increased wait; with certain times and days being recognised as particularly busy. These times, for example, included evenings, with particular reference to Friday and Saturday nights. Here it is recognised by participants that EDs are regularly frequented by individuals who are intoxicated or who have injuries associated with alcohol and illicit drug use.

Often there were negative feelings expressed about the use of EDs in relation to alcohol and drug use. In particular the view that attendance under such circumstances was a misuse of the service.

'I think they were just happy to have a call out on a Friday night that wasn't a drunk in town basically. It was just an easy job for them.' (P6, Female, Known epilepsy)

'if it was 10 o'clock on a Saturday night it would be totally different wouldn't it…' (P8, Female, First unprovoked seizure)

Finally, some participants recognised the use of the triage system, and the need to prioritise care of "*other, sicker patients*" as a cause for delays in their own treatment. More commonly with seizures, participants were conveyed to ED via ambulance. This was seen by one participant as a quicker way into ED than the "*normal*" route of self-referral to ED and attending via patient's own method of transport.

'yes the normal way I think it is longer but with the ambulance taking you I think you get seen quicker.' (P1, Female, Known epilepsy)

'it depends on what time of day you go, it depends on how you go, get taken into A&E it affects everything you know.' (P2, Female, First unprovoked seizure)

Continuity of care

Three second-order themes were defined within the main theme of continuity of care; perceptions of care continuity, accessibility of services responsive to need, and sense of abandonment.

For peer review only - http://bmjopen.bmj.com/site/about/guidelines.xhtml

Perceptions of continuity

Doctors within the ED are responsible for informing seizure patients of the subsequent stages in their care pathway. This should be a timely referral to a neurology outpatient appointment. When asked about their understanding of this process, most participants acknowledged being told about the follow-up procedure, highlighting a positive example of communication within the ED.

In the few cases where participants were not told about the pathway, or when their waiting time for follow-up was delayed, this led to concern and anxiety, particularly among first seizure patients.

Conversely, those with known epilepsy expressed lower levels of anxiety in response to lack of advisement around follow-up procedures. Such participants often demonstrate their knowledge of the system; proactively accessing services they have previously used to obtain the follow-up care they need. This finding highlights that patients with known epilepsy, when provided with appropriate means, are able to proactively seek care to address their needs.

> *'it's like we have our own pathway that just sort of made ourselves… but that sort of works because of the fact that for her condition it's easier to manage everything ourselves so we know what's going on. As far as the only pathway is when all the information gets handed over if she's got an appointment she wouldn't know what it's for. Whereas once she's booked one directly with [epilepsy specialist nurse] she knows why she's going and what she wants to talk about.'* (P6, Female, Known epilepsy)

The role of the epilepsy specialist nurse (ESN) was solely discussed by participants with known epilepsy. In this group participants spoke positively of the service and knowledge provided by the nurse specialists. As one participant noted:

> *'so [epilepsy specialist nurse] is absolutely brilliant… yes she's quite on the ball.* (P6, Female, Known epilepsy)

However, those participants who were new to the service did not demonstrate any knowledge or understanding of the ESN role or related service provision.

For peer review only - http://bmjopen.bmj.com/site/about/guidelines.xhtml

Accessibility of services to suit need

Participants experienced numerous barriers when transitioning from emergency care to follow-up ambulatory services. Important barriers were mainly organisational whereby participants frequently recounted experience of: difficulty in negotiating the appointment system, delays in receiving referral appointments, difficulty in accessing hospital/ clinic locations and related transport problems.

Some participants noted that travelling to appointments was difficult. A combination of ill-health, inability to drive due to seizures, cost of taxis, and poor public transport connections, were noted as barriers to accessing services. This led to patients being reliant on family members to support them to get to their appointments; increasing a sense of burden and dependence in patients who were otherwise independent.

Where participants were expected to attend for more than one follow-up appointment, for example where scans and further tests were required, they felt this compounded the level of inconvenience they experienced. Participants questioned the current process around 'follow-up' care provision. It was suggested, as a means to reducing the level of inconvenience experienced by patients, that appointments be combined to reduce the number of visits, or, if possible, appointments be conducted in a local district general hospital (DGH) suitable to the patient's location, thereby reducing the resources required for access.

> *'…because I can't drive now because of the epilepsy and I'm on my own with the children it's tricky, I've missed a couple of appointments at [tertiary clinic] just because it's tricky to get there. Because if I can't get anyone to give me a lift and look after the children it's hard for me to get there... it would be easier if they could come to my local doctors and do a clinic there.'* (P35, Female, Known epilepsy)

> *'I had to go back to [tertiary hospital] every day… and obviously from here it's like an hour and it was all that petrol so my dad had to take the week off work and take me back there every day before 9am which was tiring.'* (P36, Female, Known epilepsy)

Sense of abandonment

Participants reporting of follow-up care after ED attendance varied significantly, demonstrating that all patients' who had access to the pathway at ED did not necessarily

For peer review only - http://bmjopen.bmj.com/site/about/guidelines.xhtml

receive the same benefits of the intervention post-ED discharge. Frequently, and irrespective of the level of information provided at ED discharge, this lack of continuity left patients feeling abandoned. This situation occurred predominantly at two time points; immediately after discharge from ED, and following outpatient review at neurology clinic.

After discharge from ED, participants reported expecting to receive a follow-up appointment letter from the Walton Centre. In some instances, participants spoke of experiencing significant delays in receiving this appointment, or not receiving an appointment. These experiences led participants to feel anxious, expressed as not knowing where to turn for further support. In addition, participants reported being faced with feelings of uncertainty about what to do and how long to wait before seeking further help.

> *'it was just frustrating not knowing what was going on and then the fact I had to chase it up the appointment. If I hadn't chased it up three times I'd have never got the see the neurologist… I even went to the doctors [GP] and they [GP] left it up to me to chase them up and that is definitely wrong.'* (P14, Female, Not epileptic seizure)

The above quote reflects the actions of many participants within the study. Those having problems with hospital follow-up appointments often reported accessing their general practitioner (GP) for assistance. This scenario however was seen to create further frustration with many participants feeling disappointed with care offered by the GP. Not least because it was felt the GP could not deliver the level of support needed by the participant and that often clinical decisions were referred back to neurology services.

Conversely, other participants reported feeling well supported by their GP with regard to their seizures. Although it was noted that often GPs were reluctant to make decisions with regard to anti-epileptic drugs, the support and advice offered by GPs, through liaison with neurology services, had helped participants to feel aided to manage their condition without having to access emergency care.

> *'she's [GP] been brilliant, she's been amazing, really, really good… I can't complain and you know if there was a problem I could just speak to the GP or I could ring someone at [Walton Centre] that's not a problem. I know there's always somebody available if you've got concerns.'* (P35, Female, Known epilepsy)

For peer review only - http://bmjopen.bmj.com/site/about/guidelines.xhtml

The second instance where participants recounted feelings of abandonment was following their follow-up outpatient appointments. This more commonly occurred in patients who had experienced a first seizure, or a formal epilepsy diagnosis was not reached. These patients were typically discharged back to their GP after any necessary tests. When seizures continued participants were uncertain about what to do; often not seeing the GP as adequately trained to deal with such situations. This sense of abandonment was underpinned by beliefs that the service could not meet the needs of individuals. This led to frustration in relation to care, increasing anxiety and feelings of low mood within participants.

'I did take the letter in that the neurologist sent to me but all she [GP] kept doing was reading through it and typing what that letter says onto there's. She wasn't doing her own, I don't know, her own idea of what it could be or something I think she was just copying what the neurologist said because she didn't have… in my eyes she didn't have a clue in that doctors.' (P26, Male, First unprovoked seizure)

Participants repeatedly called for GPs to be better trained to deal with epilepsy and seizures. Participants believed this would enable GP's to provide appropriate, interim support while patients waited for outpatient appointments, or during times between outpatient appointments and following discharge from neurology. In one particular case; a participant diagnosed with non-epileptic attack disorder (NEAD), lack of knowledge and understanding of the specific condition by the GP was felt to have hindered further follow-up care in the primary care setting resulting in a significant negative impact on her quality of life.

> *'I don't think she [GP] really knows what it is to be honest with you or how it affects you…it's such a new thing NEAD that they don't know what it is. Like I said I've never heard of it…if you had a seizure you had epilepsy basically that's what people think isn't it… I think there needs a lot more training in it because the way it seems, because they say it's in your head and you get people saying well if it's in your head you can get rid of it.''* (P14, Female, Not epileptic seizure)

For peer review only - http://bmjopen.bmj.com/site/about/guidelines.xhtml

This sense of abandonment and not knowing where to turn in time of 'crisis', on occasion, resulted in participants reporting overuse of the ED. Returning to the ED following each seizure, although recognised as not clinically necessary, was seen by some participants as a way back into the system. As one participant explained:

> *'I mean a lot of the time obviously again they email [tertiary hospital], they email your GP to say you've been there [A&E] you know and the doctor will look at that and just go right what's going on here why has she gone to A&E again and that's when they probably bring the appointment forward but yeah it makes you more inclined definitely to go to A&E… just to get seen and not have to wait weeks on end, months on end just for an appointment that's going to take 10 minutes… at least then even if you tell them what's going on at least they know what they're doing.'* (P36, Female, First unprovoked seizure)

Discussion

To our knowledge, this is the first study that aims to evaluate the process of a seizure care pathway by exploring patient experiences and perceptions. Despite variation in care experience throughout the process, some common themes are evident.

Waiting times and patient perceived *efficiency* within the service are fundamental elements of the care process. Perceived significant wait times contributed to poorer experiences of care. These findings are broadly consistent with other studies that demonstrate that longer wait times are associated with lower levels of patient satisfaction [28-30]. In the previous 15 years emergency hospital admissions have increased by 47%, resulting in increased pressures on already struggling EDs [31]. Waiting in corridors was seen as an acceptable and somewhat 'standard' procedure to participants who had previous knowledge or experience within the ED. Some participants described feelings of self-perceived burden as a result of their seizure attendance. This finding suggests that some patients in a post-ictal state may feel that their attendance at the ED is unnecessary and that they recognise they didn't actually require the service. This is supported by a recent study conducted in a large hospital trust which concluded that the majority of people who have had seizures do not require the facilities of the ED for treatment [7].

For peer review only - http://bmjopen.bmj.com/site/about/guidelines.xhtml

There is a need to realign our pre-hospital emergency care system to help reduce avoidable hospital attendances and admissions. Reducing the number of patients unnecessarily conveyed to hospital via ambulance through better training of ambulance staff may be a way to reduce ED burden [32]. Interventions to support self-management in patients can reduce hospital admissions, allowing patients to feel empowered in decision-making surrounding their own health care needs [33, 34].

Making time for care

Highlighted as key in this study is HCPs having the *time for care.* Time spent directly with patients has been a subject on the NHS agenda in recent years, with implementation of the NHS Institute for Innovation and Improvement Releasing Time to Care- Productive Ward initiative in 2005, designed to improve patient satisfaction by increasing direct patient care by staff [35]. Positive results were seen as a consequence of the initiative, not only in improvements in patient experience, but also in clinical and safety outcomes [36, 37]. In our study, there were mixed experiences with regard to time spent directly with HCPs, with the majority reporting a positive experience, and few reporting negatively about their level of contact time. There is little evidence that the Productive Ward initiative [36] has been utilised within emergency medicine and our findings suggest that this could be a useful tool to help improve patient experience.

Discharge support and ongoing care

Poor care co-ordination resulted in the majority of participants reporting a *sense of abandonment* at some point in their journey along the care pathway. This included feeling unprepared to be discharged, lack of help and support from GPs, and poor administrative communication with regard to follow-up appointments; all of which frequently left patients feeling anxious and helpless in their ongoing ambulatory care. This finding concurs with Hesselink et al. which also reported patients feeling unprepared at time of discharge [38]. In our study, this finding demonstrates a shortfall in the effectiveness of the pathway process; highlighting potential for improvement, to better support patients on discharge from the ED and their ongoing ambulatory care support.

A large proportion of participants found the GP's support in their follow-up care to be deficient for their needs. Some GPs do not feel confident in dealing with epilepsy and

For peer review only - http://bmjopen.bmj.com/site/about/guidelines.xhtml

making medical decisions resulting in treatment delays [39, 40]. In these cases, as in our own study, patients have reported feeling frustrated and unhappy with care. Several suggestions to improve this aspect of care can be drawn from these findings. GPs have an integral role in care continuity of patients with long term conditions [41, 42]. Improvement in training of GPs in seizure management and epilepsy medications could reduce treatment delays and further ED attendances. Furthermore GPs take on added responsibility of providing a 'safety net' to try to help when communications break down between the ED and neurology services. Further to the role of the GP, the ENS role is also designed to improve continuity and accessibility to neurology services [43, 44]. The Walton Centre NHS Foundation Trust has been named an NHS Vanguard under the New Care Models scheme which means they have national support to pioneer new ways of working that benefits patients [45]. As part of this scheme, 'integrated neurology nurses' have recently been employed to bridge the gap between The Walton Centre and the community, ultimately providing a better link between the hospital, the GP, and community multi-disciplinary teams.

Our data supports the suggestion to incorporate ongoing ambulatory care services, both from GPs and nurse specialists, within the care pathway. This would likely promote care quality across the continuum of the patient journey. Patients are faced with making difficult decisions when accessing care. Such decisions are based on their own understanding about what will be of most benefit and what will improve their care. Where patients feel at a loss regarding what to do next, a return to ED to prompt continuation of care has been reported as an option for some patients. There have been various studies around reasons for re-attendance [46, 47], but there is little prior evidence to show that poor follow-up care results directly in re-attendance to the ED. However, this study demonstrates that lack of adequate follow-up and *sense of abandonment,* results in patients' re-attending ED as a means to re-accessing the neurology system, rather than ED re-attendance occurring in response to patient perceived clinical need.

This study demonstrates that the entire seizure care pathway process needs addressing to ensure smoother patient transition and to allow patients to be better supported in their ongoing care. We recognise both areas of good practice and areas where patients' suggest improvements. These improvements, however large or small, need to be achievable in the context of the NHS. Raised patient expectations, medical and technological

For peer review only - http://bmjopen.bmj.com/site/about/guidelines.xhtml

advances, and an increased scope in what can be treated [48] , alongside a call to "*reshape the health services around the needs and aspirations of patients"* [49], has somewhat changed patient rationality about what the NHS was originally set up to deliver in relation to demands now being placed on an already struggling service. How far the NHS can go to deliver the care patients demand is something that requires attention and needs to be brought into question. It is all well and good suggesting improvements but we recognise that what works for some individuals might be detrimental to others and that we need to be realistic in our suggestions for service improvement.

Strengths and limitations

Previous work in the field of seizure pathway evaluation did not qualitatively explore patient experience [13].To our knowledge this study is the first to evaluate the seizure care pathway by exploring the views of patients who have directly experienced the pathway. These findings provide a more nuanced exploration of the pathway intervention, albeit sensitive to the context is being delivered.

Participants were recruited from three NHS study sites within NW England; all had a specific research nurse responsible for embedding the care pathway and follow-up procedure. This could potentially be a limitation, as the issues identified are specific to a nurse-led care pathway. In addition, this is a local initiative and as such findings from the current study may not be generalizable to other settings/ geographical locations. Although we recognise some limitations, we have tried to make the sample as representative as possible by adopting a purposive sampling approach characteristic of the general epilepsy population. This study did not capture perspectives of staff working within the field of the seizure care pathway. We recognised this as an area likely to provide further insight around the care pathway implementation process and we are currently conducting a sub-study to qualitatively explore the views of these individuals.

Conclusions

Clinical, professional and organisational barriers all have impact on the delivery of 'complex interventions' such as that of the seizure care pathway. That said, patients rely on such interventions, along with the support of HCPs', to support their transition from ED to

For peer review only - http://bmjopen.bmj.com/site/about/guidelines.xhtml

ambulatory care. Although spanning a broad spectrum, there are various service-focused changes that have been suggested and discussed through our findings which may help to improve experience in this patient group. Refining interventions to support self-management, improved training for ambulance staff, and more efficient use of current services such as epilepsy specialist nurses and the 'integrated neurology nurses' could all prove useful in improving experience. Recognising what the NHS can and cannot deliver is vital in selecting the best ways to improve services in line with NHS values. This will not only drive quality of care for service users but also support the NHS in reducing its current financial deficits [50].

Figure Legend

Fig.1 Schematic diagram demonstrating the five main themes identified in the study, surrounding the two central and integrative themes of 'lived experience' and 'communication'.

Acknowledgements The authors would like to thank the four NHS hospital trusts for their support with recruiting participants, and those patients who gave their time to take part in the study.

Contributors TM, PD, and AN conceived the initial idea of the study, designed the trial, and obtained research funding. LM recruited, carried out, and analysed the interviews, with input from DS. LM drafted the manuscript and PD, AN, DS and TM made contributions to its development. All authors reviewed and approved the final manuscript.

Funding This work was supported by the National Institute of Health Research (CLAHRC NWC). The views expressed are those of the author(s) and not necessarily those of the NHS, the NIHR or the Department of Health.

Competing interests None declared.

Ethics approval Ethical approvals were obtained from the research sponsor (University of Liverpool - protocol number: UoL001140) and from Wales Research Ethics Committee 7, (reference number: 15/WA/0207). Approval from HRA and the Research and Development department of each of the participating NHS hospital trusts (IRAS ID 173222) was also sought prior to commencing the study.

Data sharing statement No additional data are available.

For peer review only - http://bmjopen.bmj.com/site/about/guidelines.xhtml

References

1. House of Commons Committee of Public Accounts. Financial sustainability of the NHS: Forty-third Report of Session 2016-17 2017.
2. NHS England. NHS Five Year Forward View. London: NHS England; 2014.
3. Dickson JM, Taylor LH, Shewan J, Baldwin T, Grünewald RA, Reuber M. Cross-sectional study of the prehospital management of adult patients with a suspected seizure (EPIC1). BMJ Open. 2016;6(2).
4. Dixon PA, Kirkham JJ, Marson AG, Pearson MG. National Audit of Seizure management in Hospitals (NASH): results of the national audit of adult epilepsy in the UK. BMJ Open. 2015;5(3).
5. Hiller M, Great Britain: Parliament: House of Commons: Committee of Public Accounts. HC 502 - Services to People with Neurological Conditions: Progress Review: Stationery Office; 2016.
6. Ridsdale L, Virdi C, Noble A, Morgan M. Explanations given by people with epilepsy for using emergency medical services: A qualitative study. Epilepsy Behav. 2012;25(4):529-33.
7. Dickson JM, Dudhill H, Shewan J, Mason S, Grünewald RA, Reuber M. Cross-sectional study of the hospital management of adult patients with a suspected seizure (EPIC2). BMJ Open. 2017;7(7).
8. Reuber M, Hattingh L, Goulding PJ. Epileptological emergencies in Accident and Emergency: a survey at St James's University Hospital, Leeds. Seizure. 2000;9(3):216-20.
9. Girot M, Hubert H, Richard F, Chochoi M, Deplanque D, Derambure P, et al. Use of emergency departments by known epileptic patients: An underestimated problem? Epilepsy Research. 2015;113(Supplement C):1-4.
10. National Institute for Health and Care Excellence. Epilepsies: diagnosis and management. NICE Guideline [CG137]. 2016.
11. Luciano AL, Shorvon SD. Results of treatment changes in patients with apparently drug-resistant chronic epilepsy. Annals of Neurology. 2007;62(4):375-81.
12. Dubuc N, Bonin L, Tourigny A, Mathieu L, Couturier Y, Tousignant M, et al. Development of integrated care pathways: toward a care management system to meet the needs of frail and disabled community-dwelling older people. International Journal of Integrated Care. 2013;13:e017.
13. Iyer PM, McNamara PH, Fitzgerald M, Smyth L, Dardis C, Jawad T, et al. A Seizure Care Pathway in the Emergency Department: Preliminary Quality and Safety Improvements. Epilepsy Research and Treatment. 2012.
14. Campbell M, Fitzpatrick R, Haines A, Kinmonth AL, Sandercock P, Spiegelhalter D, et al. Framework for design and evaluation of complex interventions to improve health. BMJ. 2000;321(7262):694-6.
15. Moore GF, Audrey S, Barker M, Bond L, Bonell C, Hardeman W, et al. Process evaluation of complex interventions: Medical Research Council guidance. BMJ : British Medical Journal. 2015;350.
16. Grant A, Treweek S, Dreischulte T, Foy R, Guthrie B. Process evaluations for cluster-randomised trials of complex interventions: a proposed framework for design and reporting. Trials. 2013;14(1):15.
17. Palinkas LA, Horwitz SM, Green CA, Wisdom JP, Duan N, Hoagwood K. Purposeful sampling for qualitative data collection and analysis in mixed method implementation research. Administration and policy in mental health. 2015;42(5):533-44.
18. Patton MQ. Qualitative research and evaluation methods. . Thousand Oaks, CA: Sage Publications; 2002.
19. Manzano A. The craft of interviewing in realist evaluation. Evaluation. 2016;22(3):342-60.
20. The Co-ordination Centre. NHS Emergency Department Questionnaire The Co-ordination Centre: Care Quality Commission; 2016 [cited 2017 12th September]. Available from: http://www.nhssurveys.org/Filestore/AE16/AE16_Questionnaire_v1.pdf.
21. King N. 'Doing template analysis'. In: Symon G, Cassell C, editors. Qualitative Organisational Research: Core Methods and Current Challenges. London: SAGE; 2012. p. 426-50.

For peer review only - http://bmjopen.bmj.com/site/about/guidelines.xhtml

22. NVivo qualitative data analysis Software. QSR International Pty Ltd.; 2012.
23. Brooks J, McCluskey S, Turley E, King N. The Utility of Template Analysis in Qualitative Psychology Research. Qualitative Research in Psychology. 2015;12(2):202-22.
24. Elo S, Kyngas H. The qualitative content analysis process. J Adv Nurs. 2008;62.
25. Armstrong D, Gosling A, Weinman J, Marteau T. The Place of Inter-Rater Reliability in Qualitative Research: An Empirical Study. Sociology. 1997;31(3):597-606.
26. Ritchie J, Spencer L. Qualitative data analysis in applied policy research. In: Bryman A, Burgess R, editors. Analyzing Qualitative Data. London: Routledge; 1994.
27. Pope C, Ziebland S, Mays N. Analysing qualitative data. BMJ : British Medical Journal. 2000;320(7227):114-6.
28. Anderson RT, Camacho FT, Balkrishnan R. Willing to wait?: The influence of patient wait time on satisfaction with primary care. BMC health services research. 2007;7:31-.
29. Topacoglu H, Karcioglu O, Ozucelik N, Ozsarac M, Degerli V, Sarikaya S, et al. Analysis of factors affecting satisfaction in the emergency department: A survey of 1019 patients. Advances in Therapy. 2004;21(6):380-8.
30. Bleustein C, Rothschild DB, Valen A, Valatis E, Schweitzer L, Jones R. Wait times, patient satisfaction scores, and the perception of care. The American journal of managed care. 2014;20(5):393-400.
31. National Audit Office. Emergency admissions to hospital: managing the demand. London: The Stationery Office; 2013.
32. Noble AJ, Snape D, Goodacre S, Jackson M, Sherratt FC, Pearson M, et al. Qualitative study of paramedics' experiences of managing seizures: a national perspective from England. BMJ Open. 2016;6(11).
33. Purdy S. Avoiding hospital admissions. What does the research say? : The Kings Fund; 2010.
34. Leenen LAM, Wijnen BFM, van Haastregt JCM, de Kinderen RJA, Evers SMAA, Majoie MHJM, et al. Process evaluation of a multi-component self-management intervention for adults with epilepsy (ZMILE study). Epilepsy Behav. 2017;73(Supplement C):64-70.
35. NHS Institute for Innovation and Improvement. The Productive Ward: Releasing time to care. Learning and Impact Review. Executive Summary. Coventry: NHS Institute for Innovation and Improvement; 2010.
36. Wilson G. Implementation of releasing time to care – the productive ward. Journal of Nursing Management. 2009;17(5):647-54.
37. White M. How effective is the Productive Ward? Nursing Times. 2015;111(8):22-5.
38. Hesselink G, Flink M, Olsson M, Barach P, Dudzik-Urbaniak E, Orrego C, et al. Are patients discharged with care? A qualitative study of perceptions and experiences of patients, family members and care providers. BMJ Quality & Safety. 2012;21(Suppl 1):i39-i49.
39. Thapar AK, Stott NC, Richens A, Kerr M. Attitudes of GPs to the care of people with epilepsy. Fam Pract. 1998;15.
40. Thapar AK, Roland MO. General practitioner attitudes to the care of people with epilepsy: an examination of clustering within practices and prediction of patient-rated quality of care. BMC Fam Pract. 2005;6(1):9.
41. Freeman G, Hjortdahl P. What future for continuity of care in general practice? BMJ. 1997;314.
42. Freeman G, Hughes J. Continuity of care and patient experience2011 08/03/2017.
43. Hosking PG, Duncan JS, Sander JMW. The epilepsy nurse specialist at a tertiary care hospital—improving the interface between primary and tertiary care. Seizure. 2002;11(8):494-9.
44. Goodwin M, Higgins S, Lanfear JH, Lewis S, Winterbottom J. The role of the clinical nurse specialist in epilepsy: A national survey. Seizure. 2004;13(2):87-94.
45. NHS England. New Care Models: Vanguards- developing a blueprint for the future of NHS and care services London: NHS England; 2016.

For peer review only - http://bmjopen.bmj.com/site/about/guidelines.xhtml

46. Noble AJ, Goldstein LH, Seed P, Glucksman E, Ridsdale L. Characteristics of people with epilepsy who attend emergency departments: Prospective study of metropolitan hospital attendees. Epilepsia. 2012;53(10):1820-8.
47. Allard J, Shankar R, Henley W, Brown A, McLean B, Jadav M, et al. Frequency and factors associated with emergency department attendance for people with epilepsy in a rural UK population. Epilepsy Behav. 2017;68(Supplement C):192-5.
48. Seddon N. Quite like heaven? Options for the NHS in a consumer age. London: Civitas; 2007.
49. Department of Health. The NHS Improvement Plan- Putting People at the Heart of Public Services. London: The Stationery Office; 2004.
50. Department of Health. The NHS Constitution. London: Williams Lea; 2015.

For peer review only

For peer review only - http://bmjopen.bmj.com/site/about/guidelines.xhtml

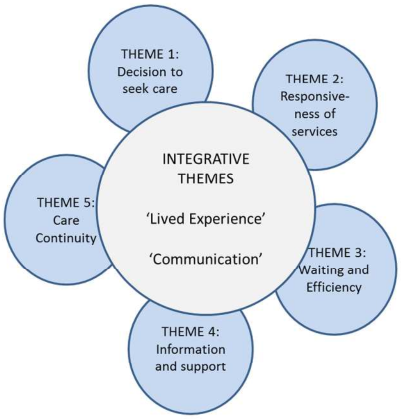

Fig.1 Schematic diagram demonstrating the five main themes identified in the study, surrounding the two central and integrative themes of 'lived experience' and 'communication'.

338x190mm (300 x 300 DPI)

review only

BMJ Open

Perceptions of emergency care using a seizure care pathway for patients presenting to emergency departments following a seizure; a qualitative study.

Journal:	*BMJ Open*
Manuscript ID	bmjopen-2017-021246.R1
Article Type:	Research
Date Submitted by the Author:	09-Apr-2018
Complete List of Authors:	Male, Leanne; University of Liverpool, Department of Neuroscience<br>Noble, Adam; University of Liverpool, Department of Psychological Sciences<br>Snape, Darlene; University of Liverpool, Department of Psychological Sciences<br>Dixon, Peter; University of Liverpool, Department of Biostatistics<br>Marson, Tony; University of Liverpool, Institute of Translational Medicine; The Walton Centre NHS Foundation Trust
**Primary Subject Heading**:	Health services research
Secondary Subject Heading:	Emergency medicine, Neurology, Qualitative research
Keywords:	ACCIDENT & EMERGENCY MEDICINE, Organisation of health services < HEALTH SERVICES ADMINISTRATION & MANAGEMENT, Quality in health care < HEALTH SERVICES ADMINISTRATION & MANAGEMENT, Epilepsy < NEUROLOGY, QUALITATIVE RESEARCH

SCHOLARONE™
Manuscripts

V10 27/03/2018

Perceptions of emergency care using a seizure care pathway for patients presenting to emergency departments following a seizure; a qualitative study.

Authors

Leanne Male *, Dr. Adam Noble *, Dr. Darlene Snape*, Dr. Peter Dixon*, Prof. Tony Marson *,¶

* University of Liverpool, UK ¶ The Walton Centre NHS Foundation Trust, Liverpool, UK

Corresponding Author

Address: Room 2.29, Clinical Sciences Centre, Aintree University Hospital, Lower Lane, Fazakerley, L9 7LJ. **Email:** hllmale@student.liv.ac.uk

Co-Authors

Dr. Adam Noble, University of Liverpool, Department of Psychological Sciences, Liverpool, UK

Dr. Darlene Snape, University of Liverpool, Department of Psychological Sciences, Liverpool, UK

Dr. Peter Dixon, University of Liverpool, Department of Biostatistics, Liverpool, UK

Prof. Tony Marson, University of Liverpool, Institute of Translational Medicine, Liverpool, UK

The Walton Centre NHS Foundation Trust, Liverpool, UK.

Word Count- 6009

Keywords

Emergency medical services, seizures, epilepsy, qualitative research, process evaluation

For peer review only - http://bmjopen.bmj.com/site/about/guidelines.xhtml

Perceptions of emergency care using a seizure care pathway for patients presenting to emergency departments following a seizure; a qualitative study.

Abstract

Objectives To establish the appropriateness of a previously developed seizure care pathway by exploring to what extent patients valued the intervention and perceived it as being helpful or not.

Design Qualitative descriptive study, using semi-structured, in-depth interviews and thematic template analysis, theoretically informed by critical realism.

Setting In North-West England, a seizure care pathway has been developed in collaboration with a specialist neurology hospital, to support clinical management of seizure patients on initial presentation to the emergency department (ED), as well as access to follow-up services on discharge, with the aim of improving patient experience. Three National Health Service EDs and a specialist neurology hospital provided the setting for participant recruitment to this study.

Participants 27 participants following their experience of an ED attendance and outpatient follow-up appointment after a seizure.

Results Five main themes emerged from the data; decision to seek care, responsiveness of services, waiting and efficiency, information and support, and care continuity. Two integrative themes spanned the whole study; lived experience and communication. This paper reports on two of the main themes; care continuity and waiting and efficiency.

Conclusions Implementation of a care pathway is a complex intervention, requiring long-term follow-up to assess its integration into practice and effectiveness in service improvement. The seizure care pathway has the potential to enhance the care of seizure patients in the ED and at follow-up by improving continuity and management of care. The study demonstrates good aspects of the seizure care pathway as observed by patients, but also recognises shortcomings within current service provision and questions what the NHS should and should not be delivering. Our study suggests various ways to enhance the pathway at service-level to potentially drive improved patient experience.

For peer review only - http://bmjopen.bmj.com/site/about/guidelines.xhtml

Strengths and limitations of this study

- This is the first in-depth study exploring patient experience of an ED seizure care pathway in the UK.
- The study allows the voices of both patients with known epilepsy and those experiencing their first seizure to be heard, as well as their carers/relatives, allowing for data to be collected from multiple viewpoints.
- Despite presenting at different NHS trusts, participants often reported similar experiences; , indicating issues reported are not site specific and do not occur in isolation, making the findings transferable between trusts.
- The study did not capture the perspectives of staff regarding the delivery of the care pathway in practice, which may give further insight into the appropriateness and effectiveness of its use.
- It remains to be established whether addressing the issues raised by participants about their experience of the pathway would translate into improved care quality and patient experience.

For peer review only

For peer review only - http://bmjopen.bmj.com/site/about/guidelines.xhtml

V10 27/03/2018

Introduction

The NHS, like other health systems around the world, is operating within a context of rising demand, slow growth in funding and increasing operating costs. As a consequence, NHS providers ended 2015/16 with a funding deficit of £2.45 billion [1]. The Five Year Forward View challenges the NHS to make unprecedented savings [2]. One way to achieve this is to reduce avoidable use of expensive emergency hospital services.

In the UK, seizures account for 3.3% of all calls to the emergency ambulance service [3], ~60,000 seizure-related ED attendances and ~40,000 hospital admissions each year, at a cost to the NHS in 2015/16 of ~£70 million [4-6]. Emergency care for seizures can be appropriate, important, and even life-saving. Despite this, evidence suggests that seizure patients' attendance at the emergency department (ED) is, more often than not, clinically unnecessary [7-9].

Frequently, seizure-related ED visits are though expressions of sub-optimal ambulatory care. The National Audit of Seizure management in Hospitals (NASH) found that most of those presenting to the ED with known epilepsy (~65%) had not been seen an epilepsy specialist within the past 12 months, but were in need of such support. They were often treated using sub-optimal, outdated drug treatment regimens and so at risk of avoidable seizures and/ or adverse events [4]. NICE (2016) recommend when seizures are not controlled or treatment fails, the patient should be referred to a specialist for assessment [10]. Evidence suggests such input could help improve patient outcomes [11]. ED visits do not, however, currently lead to most attendees receiving such specialist support [4].

Part of the solution might lie with the introduction of a clinical care pathway within acute hospitals. Care pathways have proven to work in the NHS as a means of directing and co-ordinating patient care across health care providers [12]. Importantly, a recent study in the Republic of Ireland saw the implementation of a pathway specifically for seizures. It was designed to improve clinical management, reduce unnecessary admissions, minimise length of stay following ED attendance and improve follow-up services through a rapid access clinic [13]. Initial evidence suggests the pathway has utility in reducing length of stay for patients and time until the patient is followed-up by outpatient services [13].

Ensuring equitable access to specialist neurological care in the UK has long been challenging. The above evidence suggests instituting a more proactive and better

For peer review only - http://bmjopen.bmj.com/site/about/guidelines.xhtml

coordinated model of care may hold promise in driving up care quality while at the same time reducing emergency service use for seizures. Within the Cheshire and Mersey region of England, a seizure care pathway was therefore developed. Seizure services in the area had been identified as a key target for improvement and better co-ordination [4]. The Cheshire and Merseyside Strategic Clinical Network (SCN) co-ordinated the development of the pathway for adults presenting to emergency services with seizures in collaboration with stakeholders (neurologists, general practitioners, patient and public representatives and commissioners).

The pathway constitutes a paper proforma which provides clinical advice on patient management and investigation, and instructions as to next steps and how to access the neurology/seizure service. Patients should be made aware of the seizure care pathway, offered an advice leaflet, be informed that they will be referred to the Walton Centre NHS Foundation Trust which is the region's tertiary neuroscience hospital (and to expect to hear about an appointment in due course. Following patient ED assessment, the proforma is faxed within 24 hours to the Walton Centre appointments office for processing. Patients will then be contacted by telephone to organise a neurology out-patient appointment in a time appropriate manner; ideally within 2 weeks of their ED attendance, as per NICE guidance [10].

The Care After Presenting with Seizures (CAPS) study, funded by the National Institute of Health Research (CLAHRC NWC), was set up to evaluate the pathway's effect. CAPS is a non-randomised trial comparing the effect of the pathway to usual care alone in reducing the number of emergency visits made by patients with seizures attending three local hospital EDs .

Government policy has, for a number of years, promoted the encouragement of patients to contribute to the planning and development of health care services as a means to providing them with a stronger voice in relation to decisions around their own health and care and to facilitate service development that reflects service-user needs and preferences [2, 14]. The Five Year Forward View outlines the need for change in the relationship between people and the NHS; empowering patients to be actively involved in their own care, including working collaboratively on the development of health service delivery plans [2]. Such active patient involvement has been shown to improve outcomes and experiences for patients [15]. Whilst the seizure care pathway has been designed with patient involvement, it

For peer review only - http://bmjopen.bmj.com/site/about/guidelines.xhtml

remains important to continue this involvement following implementation of the pathway in clinical practice, to help ensure it meets patients' needs, as well as clinical requirements.

This paper presents the findings of a qualitative study of patient experiences' of the seizure care pathway. Specifically, it aimed to explore: 1) whether the pathway was valued by patients and, if so, why?; and, (2) what aspects of the intervention were perceived by patients as being helpful or unhelpful and why.

Methods

Study Design and Setting

This work is a qualitative study conducted with semi-structured patient interviews. We were interested in a descriptive evaluation of the seizure care pathway based on participants' understanding, perceptions and experiences, both in relation to their ED care following a seizure presentation and the subsequent follow-up service offered.

Participants

Table 1 outlines the inclusion/exclusion criteria for the study. Screening was undertaken by LM, a PhD student with a nursing background.

Table 1
Participant inclusion/exclusion criteria for study

Inclusion Criteria	Exclusion Criteria
• Aged 16 years or older.<br>• Presented in ED following a suspected seizure, regardless of whether they had a previous clinical diagnosis of epilepsy.	• Provoked seizure (e.g. alcohol withdrawal).<br>• Event considered unlikely to be an unprovoked seizure (e.g. syncope).<br>• Reside outside of Walton Centre's catchment area<br>• Learning disability.<br>• Not fluent in written or spoken English.

Participants were recruited using purposive sampling, as detailed in the results section, to allow representation of the diverse range of patients attending the ED following a seizure [16, 17].

For peer review only - http://bmjopen.bmj.com/site/about/guidelines.xhtml

V10 27/03/2018

Patient and Public Involvement

Patient involvement began at the outset during the design stages of the study; development of the topic guide and research questions. The project was supported by a local charity where public support was received and utilised in the form of recruiting pilot interviewees and support with topic guide development through discussion and sharing of ideas around patient preferences, priorities and previous experiences.

Results will be disseminated to participants through a lay summary of findings posted out to all participants following the analysis of all interviews.

For peer review only

Data Collection

Following ethical approval from Wales Research Ethics Committee (Ref. 15/WA/0207), ostensibly eligible patients were contacted to discuss the study further and, if interested, a time and date for interview was arranged. Participant consent was obtained prior to commencement of the interview. Face-to-face interviews were conducted with the participant and, if requested by the participant, a carer - who had been present at the time of the ED attendance – was also invited to contribute. Interviews were audio recorded and transcribed verbatim.

The interview topic guide (Table 2) was developed through a review of the relevant literature. The topic guide was piloted and iterative data analysis, researcher reflections and study team discussions were utilised to update the topic guide throughout the process of data collection [18].

Table 2
Specific topics introduced within interviews.

Figure 1. Topic guide for interviews
Can you tell me about what happened to make you attend ED?<br>Probe: experience, decision-making, transport to ED.
Tell me what you remember about your experience in the ED.<br>Probe: Care processes, interactions with professionals, involvement in decision-making, information and support provision.
Was the most recent experience any different to past experiences of ED?

Probe: similarities/differences, likes/dislikes, changes.
What does 'good care' mean to you?<br>Probe: expectations, shortfalls.<br>What sort of changes, if any, could be made to improve your care throughout different stages in the ED?
Tell me about your experience of the transition of care between ED and the Walton Centre?<br>Probe: communication, timeliness, similarities/differences to previous referrals.
Can you tell me about your experience of when you went for your outpatient clinic appointment?<br>Probe: usefulness of appointment, benefits of pathway, improvement suggestions, how well do you feel your 'crisis' was dealt with?
Discuss the usefulness of the current ED Questionnaire (Picker Institute) from patients' opinion[19].

Data analysis

Interviews were analysed thematically using the template method, informed by the work of King [20]. QSR International's NVivo V.10 software [21] was used to facilitate data management and document the analytical process. The analytic template was developed using a combination of *a priori* codes, derived from previous research around patient experience in general, and salient findings from early participant interviews. Using *a priori* codes ensured a focus on key areas potentially relevant to the study [22]. Further codes and themes were identified deductively through analysis of raw data, allowing identification of themes and patterns related to the study aims [23].

The initial template was developed by LM and another researcher (DS). Transcripts were coded separately by LM and DS before comparison was made using the coding template [24]. Transcripts were read and re-read, further amendments were made to the template until coding consensus was reached [25, 26]. Final themes were reviewed by the study team to ensure they addressed the subject matter and accounted for the data content.

Findings

Participant characteristics

For peer review only - http://bmjopen.bmj.com/site/about/guidelines.xhtml

Participants who met the inclusion criteria for CAPS, and had received a follow-up appointment following their ED visit, were invited to participate in the qualitative study. One hundred and eighty-one patients were screened for eligibility and contacted via telephone to invite them to take part in the study. Of these, 17 patients declined to participate. Reasons given for their refusal included anxiety, poor memory, and time constraints. A further 137 potential participants could not be accessed due to patients not answering telephone calls/ not replying to messages left, and incorrect patient contact details.

The overall acceptance rate was therefore 22%, with 15% of those screened eventually participating. A total of twenty seven interviews were conducted between March 2016 and May 2017. Twenty five of the interviews were conducted in the participant's own home, with the remaining two interviews, taking place at an alternative location at the request of the participant. The average time between participants' ED presentations and interviews was 100 days (SD 32.6). Demographics and clinical characteristics of the study sample are detailed in Table 3.

Table 3
Patient demographics and clinical characteristics, grouped according to diagnosis following neurology review.

	Known Epilepsy (n=13)	First unprovoked Seizure (n= 8)	Not epileptic Seizure (n=6)
**Age (years)**			
-Mean	50	37	32
-Range	24-78	17-64	19-45
**Gender**			
-Male	6 (46.2%)	5 (62.5%)	4 (66.7%)
-Female	7 (53.8%)	3 (37.5%)	2 (33.3%)
**Employment**			
-Full time work	3 (23.1%)	1 (12.5%)	2 (33.3%)
-Part time work	2 (15.4%)	1 (12.5%)	0 (0%)
-Not employed	8 (61.5%)	5 (62.5%)	3 (50%)
-Education	0 (0%)	1 (12.5%)	1 (16.7%)
**IMD Regional Decile**			
-1-3 (most deprived)	9 (69.2%)	4 (50%)	3 (50%)
-4-6	2 (15.4%)	3 (37.5%)	2 (33.3%)
-7-10 (least deprived)	2 (15.4%)	1 (12.5%)	1 (16.7%)

For peer review only - http://bmjopen.bmj.com/site/about/guidelines.xhtml

Identified themes

Analysis of the transcripts elicited five key themes (Figure 1). In addition, two integrative concepts: 'Lived Experience' and 'Communication' permeated participant discussions and as such were common across all themes whatever the foreground; proving important throughout the holistic analytic process.

The present paper focuses on findings from two of the five top level themes; namely, 'waiting and efficiency' and 'continuity of care'. Care continuity, both within the ED and across services, was highlighted as the most important to participants in relation to their overall experience of the care pathway, along with waiting, which was frequently referred to throughout interviews. Each theme/sub-theme will be discussed in turn in relation to participants' perception of the pathway as an effective intervention and examine each in relation to its perceived value to the pathway process overall.

Waiting and efficiency

Time was viewed by most participants as a factor influencing experience. Irrespective of whether the participant perceived their treatment as being fast or slow, time was continually mentioned, highlighting its importance in the process experienced by participants.

Participants' experience of waiting times varied at different points in their journey through the ED. Reference was repeatedly made to '*waiting for triage*', '*waiting for tests*' and '*waiting to see a doctor*'. When participants perceived their wait time as being '*quick'*, particularly in comparison to previous experience, they reported positive feedback on the process.

> *'there was no question of she's going to wait or whatever; come right through and I say I saw that doctor…they were absolutely brilliant I couldn't fault them at all.'* (P8, Female, First unprovoked seizure)

Conversely, participants perceived 'long' waiting contributed to poor experience.

For peer review only - http://bmjopen.bmj.com/site/about/guidelines.xhtml

> *'if your waiting on one of them beds to see a doctor…it does make you feel a bit on edge…people just get upset sitting out there.'* (P29, Male, Known epilepsy)

Waiting and environmental resources

Participants frequently described the physical environment of the ED as "*busy*". A commonly reported feature was lack of bed space within the department, resulting in participants being left on trollies in the ED corridor for significant periods of time. As one female explained:

> *'they were so backed up that we came in via ambulance and you know the way they have all the trolleys down the corridor backed up, they were that bad that the paramedics had to stay with me for 3 hours in a corridor.'* (P5, Female, Known epilepsy)

Similarly, this scenario had a significant impact on another participant's ED experience, prompting his self-discharge from the service. In this instance, the participant reported being in a post-ictal state but felt he no longer particularly required treatment; a decision informed, in part, by the belief he was a burden to the paramedics who had to remain with him during his time on a trolley in the corridor. He explained:

> *'there was the guilt trip I suppose on the last one because I had, this was adding to the anxiety because I was stuck on a trolley with paramedics looking after me and I'm thinking they could be out saving someone else's life. So that added to the pressure if that makes sense. Erm and that's why I said look you can't wait any longer and so I signed a self-discharge form because I felt guilty.'* (P32, Male, Known epilepsy)

Further interviews revealed participants' observations about the environment (often described as "*busy*" and "*noisy*") as well as their views about how it could be improved in a way that was more conducive to supporting those following a seizure. Suggestions for side rooms, dimmed lighting, and a quieter area to enable patients to recover from their seizure in a calm and timely manner were articulated.

> *'…they realised then they had to put me in a dark room to take my bloods and everything and then they said we'll try and put you somewhere quiet… they moved me up to the next waiting area… again there was the television on, flashing lights*

For peer review only - http://bmjopen.bmj.com/site/about/guidelines.xhtml

and we said you know you can't do that. If you put me in a dark room where it is nice and quiet my seizures will stop and calm down but they won't listen.' (P5, Female, Known epilepsy)

Time for care

Participants not only spoke about time in relation to waiting, but also with reference to time health care professionals (HCPs) spent engaging with them. While the majority of participants reported being satisfied with the time spent with HCPs, the visibility of staff members, and the communication they received, others spoke of negative experiences related to the limited time spent specifically with HCPs.

Participants reported that waiting for a doctor to arrive often induced fear and uncertainty; triggering worrying thoughts about what might be wrong and what would happen next. This feeling was articulated mainly by those participants experiencing a first seizure, for whom the event and ED environment was largely unfamiliar. Overall, participants commonly held the view that doctors and nurses were "*in a rush*". Such observations and experiences led to a sense of "*urgency*", felt by participants and a perceived inadequacy in support offered by HCPs.

'I don't know how long the doctor was with me or the nurse but it didn't seem very long at all… yes I would have liked to have known more and the amount of time I spend in there, it seemed a very little time I spent with the doctor or the nurse.' (P12, Male, Known epilepsy)

Those with known epilepsy tended to say they were more relaxed about the visibility of HCPs, not least because they expressed being more *"in control"* of the situation. More often than not, these participants reported a more positive experience of visibility of HCPs and were contented with the amount of time spent in consultation with doctors and nurses; even if this time was minimal and contact related mainly to routine basic observations.

'we were pretty much left to our own devices really, they checked that she was ok and then off they went. They weren't really regular check-ups because its more just waiting for her to come back round and be lucid and waiting for a doctor to turn up.' (P6, Female, Known epilepsy, Carer)

For peer review only - http://bmjopen.bmj.com/site/about/guidelines.xhtml

In contrast, a female with known epilepsy expressed great concern about the lack of contact with HCP's. Her concern led to fears for safety and a sense of heightened insecurity about the level of care received. These fears occasioned a negative perception of the care pathway and general experience within the ED.

> *'there was no medical staff available because they were so busy… I felt in danger because nobody knew what they were doing.'* (P5, Female, Known epilepsy)

Conceptualisation of service provision

Despite acknowledging shortfalls in the service provided, often participants expressed understanding as to why this may be the case. Most commonly, limited staff and resources in the ED were highlighted as a potential cause of increased waiting times.

> *'it was just queue of trollies with people in it. It was 4 and a half/5 hours just sat, lying around waiting… I didn't see anyone I was just lying there.'* (P26, Male, First unprovoked seizure)

Participants also noted 'time of day' at which they attended the ED as a possible causal factor for increased wait; with certain times and days being recognised as particularly busy. These times included evenings, with particular reference to Friday and Saturday nights. At these times participants said they believed EDs are regularly frequented by individuals who are intoxicated or who have injuries associated with alcohol and illicit drug use.

> *'it depends on what time of day you go, it depends on how you go, get taken into A&E it affects everything you know.'* (P2, Female, First unprovoked seizure)

> *'if it was 10 o'clock on a Saturday night it would be totally different wouldn't it…'* (P8, Female, First unprovoked seizure)

Often there were negative feelings expressed about the use of EDs in relation to alcohol and drug use. In particular the view that attendance under such circumstances represented service misuse.

For peer review only

For peer review only - http://bmjopen.bmj.com/site/about/guidelines.xhtml

'I think they were just happy to have a call out on a Friday night that wasn't a drunk in town basically. It was just an easy job for them.' (P6, Female, Known epilepsy)

Finally, some participants recognised the use of the triage system, and the need to prioritise care of "*other, sicker patients*" as a cause for delays in their own treatment. Participants with seizures had typically been conveyed to ED via ambulance. This was seen by one participant as a quicker way into ED than the "*normal*" route of self-referral to ED and not attending by ambulance.

'yes the normal way I think it is longer but with the ambulance taking you I think you get seen quicker.' (P1, Female, Known epilepsy)

Continuity of care

Three second-order themes were defined within the main theme of continuity of care; perceptions of care continuity, accessibility of services responsive to need, and sense of abandonment.

Perceptions of continuity

According the pathway proforma, the patients attending doctor within the ED is responsible for informing the patient of the subsequent stages in their care pathway. This should be a timely referral for a neurology outpatient appointment. When asked about their understanding of this process, most participants acknowledged being told about the follow-up procedure, highlighting a positive example of communication within the ED.

In the few cases where participants were not told about the pathway, or when their waiting time for follow-up was delayed, this led to concern and anxiety, particularly among first seizure participants.

Conversely, those with known epilepsy expressed lower levels of anxiety in response to lack of advisement around follow-up procedures. Such participants often demonstrated their knowledge of the health service system; proactively accessing services they have previously used to obtain the follow-up care they wanted. This finding highlights that patients with known epilepsy, when provided with appropriate means, are able to proactively seek care to address their needs.

For peer review only - http://bmjopen.bmj.com/site/about/guidelines.xhtml

'it's like we have our own pathway that just sort of made ourselves… but that sort of works because of the fact that for her condition it's easier to manage everything ourselves so we know what's going on. As far as the only pathway is when all the information gets handed over if she's got an appointment she wouldn't know what it's for. Whereas once she's booked one directly with [epilepsy specialist nurse] she knows why she's going and what she wants to talk about.' (P6, Female, Known epilepsy)

Epilepsy specialist nurses (ESN) were only mentioned by participants with known epilepsy. In this group participants spoke positively of the role and knowledge of nurse specialists. As one participant noted:

'so [epilepsy specialist nurse] is absolutely brilliant… yes she's quite on the ball. (P6, Female, Known epilepsy)

However, those participants who were new to the service did not demonstrate any knowledge or understanding of the ESN role or related service provision.

Accessibility of services to suit need

Participants experienced numerous barriers when transitioning from emergency care to follow-up ambulatory services. Important barriers were mainly organisational whereby participants frequently recounted experience of: difficulty in negotiating the appointment system, delays in receiving referral appointments, difficulty in accessing hospital/ clinic locations and related transport problems.

Some participants noted that travelling to appointments was difficult. A combination of ill-health, inability to drive due to seizures, cost of taxis, and poor public transport connections, were noted as barriers to accessing services. This led to patients being reliant on family members to support them to get to their appointments; increasing a sense of burden and dependence in patients who were otherwise independent.

Where participants were expected to attend for more than one follow-up appointment, for example where scans and further tests were required, they felt this compounded the level of inconvenience they experienced. Participants questioned the current process around 'follow-up' care provision. It was suggested, as a means to reducing the level of inconvenience experienced by patients, that appointments be combined to reduce the number of visits, or, if possible, appointments be conducted in a local district

For peer review only - http://bmjopen.bmj.com/site/about/guidelines.xhtml

general hospital (DGH) suitable to the patient's location, thereby reducing the resources required for access.

> *'…because I can't drive now because of the epilepsy and I'm on my own with the children it's tricky, I've missed a couple of appointments at [tertiary clinic] just because it's tricky to get there. Because if I can't get anyone to give me a lift and look after the children it's hard for me to get there... it would be easier if they could come to my local doctors and do a clinic there.'* (P35, Female, Known epilepsy)

> *'I had to go back to [tertiary hospital] every day… and obviously from here it's like an hour and it was all that petrol so my dad had to take the week off work and take me back there every day before 9am which was tiring.'* (P36, Female, Known epilepsy)

Sense of abandonment

Participants reporting of follow-up care after ED attendance varied significantly, demonstrating that all patients' who had access to the pathway at ED did not necessarily receive the same benefits of the intervention post-ED discharge. Frequently, and irrespective of the level of information provided at ED discharge, this lack of continuity left patients feeling *"abandoned"*. This situation occurred predominantly at two time points; immediately after discharge from ED, and following outpatient review at neurology clinic.

After discharge from ED, participants reported expecting to receive a follow-up appointment letter from the Walton Centre. In some instances, participants spoke of experiencing significant delays in receiving this appointment, or not receiving an appointment. These experiences led participants to feel anxious, expressed as not knowing where to turn for further support. In addition, participants reported being faced with feelings of uncertainty about what to do and how long to wait before seeking further help.

> *'it was just frustrating not knowing what was going on and then the fact I had to chase it up the appointment. If I hadn't chased it up three times I'd have never got the see the neurologist… I even went to the doctors [GP] and they [GP] left it up to me to chase them up and that is definitely wrong.'* (P14, Female, Not epileptic seizure)

The above quote reflects the actions of many participants within the study. Those having problems with hospital follow-up appointments often reported accessing their general practitioner (GP) for assistance. This scenario however was seen to create further

For peer review only - http://bmjopen.bmj.com/site/about/guidelines.xhtml

frustration with participants often feeling disappointed with GP care. Not least because it was felt the GP could not deliver the level of support needed by the participant and that clinical decisions were often referred back to neurology services.

Conversely, some participants reported feeling well supported by their GP with regard to their seizures. Although it was noted that GPs were often reluctant to make decisions with regard to anti-epileptic drugs, the support and advice offered by GPs, through liaison with neurology services, had helped some participants feel aided to manage their condition without having to access emergency care.

> *'she's [GP] been brilliant, she's been amazing, really, really good… I can't complain and you know if there was a problem I could just speak to the GP or I could ring someone at [Walton Centre] that's not a problem. I know there's always somebody available if you've got concerns.'* (P35, Female, Known epilepsy)

The second juncture when participants recounted feelings of abandonment was following their follow-up outpatient appointments. This more commonly occurred in patients who had experienced a first seizure, or a formal epilepsy diagnosis was not reached. These patients were typically discharged back to their GP after any necessary tests. When seizures continued participants were uncertain about what to do; often not seeing the GP as adequately trained to deal with such situations. This sense of abandonment was underpinned by beliefs that the service could not meet the needs of individuals. This led to frustration in relation to care, increasing anxiety and feelings of low mood within participants.

'I did take the letter in that the neurologist sent to me but all she [GP] kept doing was reading through it and typing what that letter says onto there's. She wasn't doing her own, I don't know, her own idea of what it could be or something I think she was just copying what the neurologist said because she didn't have… in my eyes she didn't have a clue in that doctors.' (P26, Male, First unprovoked seizure)

Participants repeatedly called for GPs to be better trained to deal with epilepsy and seizures. Participants believed this would enable GP's to provide appropriate, interim support while patients waited for outpatient appointments, or during times between

For peer review only - http://bmjopen.bmj.com/site/about/guidelines.xhtml

outpatient appointments and following discharge from neurology. In one particular case; a participant diagnosed with non-epileptic attack disorder (NEAD), lack of knowledge and understanding of the specific condition by the GP was felt to have hindered further follow-up care in the primary care setting resulting in a significant negative impact on her quality of life.

> *'I don't think she [GP] really knows what it is to be honest with you or how it affects you…it's such a new thing NEAD that they don't know what it is. Like I said I've never heard of it…if you had a seizure you had epilepsy basically that's what people think isn't it… I think there needs a lot more training in it because the way it seems, because they say it's in your head and you get people saying well if it's in your head you can get rid of it.''* (P14, Female, Not epileptic seizure)

This sense of abandonment and not knowing where to turn in time of 'crisis', on occasion, resulted in participants reporting overuse of the ED. Returning to the ED following each seizure, although recognised as not clinically necessary, was seen by some participants as a way back into the system. As one participant explained:

> *'I mean a lot of the time obviously again they email [tertiary hospital], they email your GP to say you've been there [A&E] you know and the doctor will look at that and just go right what's going on here why has she gone to A&E again and that's when they probably bring the appointment forward but yeah it makes you more inclined definitely to go to A&E… just to get seen and not have to wait weeks on end, months on end just for an appointment that's going to take 10 minutes… at least then even if you tell them what's going on at least they know what they're doing.'* (P36, Female, First unprovoked seizure)

Discussion

To our knowledge, this is the first study to explore patient experiences and perceptions of an ED seizure care pathway. Despite variation in care experience throughout the process, some common themes were evident.

Waiting times and patient perceived *efficiency* within the service are fundamental elements of the care process. Wait times that were perceived to be significant contributed to poorer experiences of care. These findings are broadly consistent with other studies that

For peer review only - http://bmjopen.bmj.com/site/about/guidelines.xhtml

demonstrate longer waits are associated with lower levels of patient satisfaction [27-29]. In the previous 15 years emergency hospital attendances have increased by 47%, resulting in increased pressures on EDs [30]. Waiting in corridors was seen as an acceptable and somewhat 'standard' procedure to participants who had previous knowledge or experience within the ED.

Some participants described feelings of self-perceived burden as a result of their seizure attendance. This finding suggests that some patients in a post-ictal state may feel that their attendance at the ED is unnecessary and that they recognise they didn't actually require the service. This is supported by a recent study conducted in a large hospital trust which concluded that the majority of people who have had seizures do not require the facilities of the ED for treatment [7].

There is a need to realign our pre-hospital emergency care system to help reduce avoidable hospital attendances and admissions. Reducing the number of patients unnecessarily conveyed to hospital via ambulance through better training of ambulance staff may be a way to reduce ED burden [31]. Interventions to support self-management in patients have the potential to reduce hospital attendances, allowing patients to feel empowered in decision-making surrounding their own health care needs [32, 33].

Making time for care

Highlighted as key in this study is HCPs having the *time for care.* Time spent directly with patients has been a subject on the NHS agenda in recent years, with implementation of the NHS Institute for Innovation and Improvement's *Releasing Time to Care- Productive Ward* initiative in 2005, designed to improve patient satisfaction by increasing direct patient care by staff [34]. Positive results were seen as a consequence of the initiative, not only in improvements in patient experience, but also in clinical and safety outcomes [35, 36]. In our study, there were mixed experiences with regard to time spent directly with HCPs, with the majority reporting a positive experience, and few reporting negatively about their level of contact time. There is little evidence that the *Productive Ward* initiative [35] has been utilised within emergency medicine and our findings suggest that this could be a useful tool to help improve patient experience.

Discharge support and ongoing care

For peer review only - http://bmjopen.bmj.com/site/about/guidelines.xhtml

Poor care co-ordination resulted in the majority of participants reporting a *sense of abandonment* at some point in their journey along the care pathway. This included feeling unprepared to be discharged, lack of help and support from GPs, and poor administrative communication with regard to follow-up appointments; all of which frequently left patients feeling anxious and helpless in their ongoing ambulatory care. This finding concurs with findings from the wider literature, with Hesselink et al. reported ED patients in general can feel unprepared at time of discharge [37]. In our study, this finding demonstrates a shortfall in the effectiveness of the pathway process; highlighting potential for improvement, to better support patients on discharge from the ED and their ongoing ambulatory care support.

A large proportion of participants felt the GP's support in their follow-up care to be deficient for their needs. Some GPs do not feel confident in dealing with epilepsy and making medical decisions resulting in treatment delays [38, 39]. In these cases, as in our own study, patients have reported feeling frustrated and unhappy with care. Several suggestions to improve this aspect of care can be drawn from these findings. GPs have an integral role in care continuity of patients with long term conditions [40, 41]. Improvement in training of GPs in seizure management and epilepsy medications could reduce treatment delays and further ED attendances. Furthermore, GPs could take on added responsibility of providing a 'safety net' to try to help when communications break down between the ED and neurology services.

Further to the role of the GP, the ENS role is also designed to improve continuity and accessibility to neurology services [42, 43]. The Walton Centre has been named an NHS Vanguard under the New Care Models scheme which means they have national support to pioneer new ways of working that benefits patients [44]. As part of this scheme, 'integrated neurology nurses' have recently been employed to bridge the gap between the centre and the community, aim to provide a better link between the hospital, the GP, and community multi-disciplinary teams.

Our findings support the suggestion of incorporating ongoing ambulatory care services, both from GPs and nurse specialists, within the care pathway. This would likely promote care quality across the continuum of the patient journey. Patients are faced with making difficult decisions when accessing care. Such decisions are based on their own understanding about what will be of most benefit and what will improve their care. Where

For peer review only - http://bmjopen.bmj.com/site/about/guidelines.xhtml

patients feel at a loss regarding what to do next, a return to ED to prompt continuation of care has been reported as an option for some patients. There have been various studies around reasons for re-attendance [45, 46], but there is little prior evidence showing that poor follow-up care results directly in re-attendance to the ED. However, this study demonstrates that lack of adequate follow-up and *sense of abandonment,* can result in some patients' re-attending ED as a means to re-accessing the neurology system.

This present study also demonstrates that the entire seizure care pathway process needs addressing to ensure smoother patient transition and to allow patients to be better supported in their ongoing care. We recognise both areas of good practice and areas where patients' suggest improvements. These improvements, however large or small, need to be achievable in the context of the NHS. Raised patient expectations, medical and technological advances, and an increased scope in what can be treated [47] , alongside a call to "*reshape the health services around the needs and aspirations of patients"* [48], has somewhat changed patient perception of what the NHS was originally set up to deliver in relation to demands now being placed on an already struggling service. The capacity of NHS service delivery needs to be taken into consideration when reviewing suggestions made for service improvement in order to make changes that will benefit patients and staff, as well as being both clinically and financially achievable.

Strengths and limitations

Previous work in the field of seizure pathway evaluation did not qualitatively explore patient experience [13]. Our findings thus provide a more nuanced exploration of the pathway intervention, albeit sensitive to the context in which it is being delivered. Interviews were conducted in the patient's location of choice. This not only advantaged recruitment as the location was more convenient for participants but also facilitated building rapport, as a non-clinical, more relaxed environment potentially encouraged participants to talk openly about their experiences. The Walton Centre catchment area is drawn from Merseyside, Cheshire, Lancashire, Greater Manchester, the Isle of Man and North Wales. The patient demographics are representative of the ED catchment area populations, where we would expect to see higher levels of unemployment and participants more likely to be living in the more deprived areas of the city.

For peer review only - http://bmjopen.bmj.com/site/about/guidelines.xhtml

Participants were recruited from three NHS study sites within NW England; all had a specific research nurse responsible for embedding the care pathway and follow-up procedure. This could potentially be a limitation, as the issues identified are specific to a nurse-led care pathway. In addition, this is a local initiative and as such findings from the current study may not be generalizable to other settings/ geographical locations.

Added to this, participant uptake was low (i.e., 22%). This figure may reflect a number of challenges associated with the conduct of this study including, for example, the chosen recruitment strategy, the difficulty in contacting patients by telephone and, the nature of epilepsy as an illness. It is important to also acknowledge there is an inherent bias with studies of this nature; those opting to participate may have strong positive or negative feelings about the service which could potentially bias the results.

The average time latency of 100 days from ED attendance to interview may also have influenced the amount of information participants could remember about their experience. While this could not be avoided as participants had to have had their outpatient appointment before being interviewed, therefore experiencing the whole pathway, it should be recognised as a potential limitation to the findings. .

This study did not capture perspectives of staff working within the field of the seizure care pathway. Previous quantitative work has though been conducted around staff perceptions of the barriers to implementation of a care pathway for seizure patients in Ireland [49] We recognise this as an important area likely to provide further insight around the care pathway implementation process in our study and are currently conducting a sub-study to qualitatively explore the views of these individuals.

Conclusions

Clinical, professional and organisational barriers all have impact on the delivery of complex interventions such as that of the seizure care pathway. That said, patients rely on such interventions, along with the support of HCPs', to support their transition from ED to ambulatory care. Although spanning a broad spectrum, there are various service-focused changes that have been suggested and discussed through our findings which may help to improve experience in this patient group. Refining interventions to support self-management, improved training for ambulance staff, and more efficient use of current

For peer review only - http://bmjopen.bmj.com/site/about/guidelines.xhtml

services such as epilepsy specialist nurses and the ‘integrated neurology nurses’ could all prove useful in improving experience. It is crucial to the health service to take into consideration the suggestions made by patients in order to drive forward care based on what patients want. However, recognising the scope of the NHS, its values, and the current financial challenges, it is important to recognise service improvements that will have the most impact. This will not only drive quality of care for service users but also potentially support the NHS in reducing its current financial deficits [50].

For peer review only

Figure Legend

Fig.1 Schematic diagram demonstrating the five main themes identified in the study, surrounding the two central and integrative themes of ‘lived experience’ and ‘communication’.

Acknowledgements The authors would like to thank the four NHS hospital trusts for their support with recruiting participants, patient advisers who supported the development of the study and those patients who gave their time to take part.

Contributors TM, PD, and AN conceived the initial idea of the study, designed the trial, and obtained research funding. LM recruited, carried out, and analysed the interviews, with input from DS. LM drafted the manuscript and PD, AN, DS and TM made contributions to its development. All authors reviewed and approved the final manuscript.

Funding This work was supported by the National Institute of Health Research (CLAHRC NWC). The views expressed are those of the author(s) and not necessarily those of the NHS, the NIHR or the Department of Health.

Competing interests None declared.

Ethics approval Ethical approvals were obtained from the research sponsor (University of Liverpool - protocol number: UoL001140) and from Wales Research Ethics Committee 7, (reference number: 15/WA/0207). Approval from HRA and the Research and Development department of each of the participating NHS hospital trusts (IRAS ID 173222) was also sought prior to commencing the study.

Data sharing statement No additional data are available.

References

For peer review only - http://bmjopen.bmj.com/site/about/guidelines.xhtml

1. House of Commons Committee of Public Accounts. Financial sustainability of the NHS: Forty-third Report of Session 2016-17 2017.
2. NHS England. NHS Five Year Forward View. London: NHS England; 2014.
3. Dickson JM, Taylor LH, Shewan J, Baldwin T, Grünewald RA, Reuber M. Cross-sectional study of the prehospital management of adult patients with a suspected seizure (EPIC1). BMJ Open. 2016;6(2).
4. Dixon PA, Kirkham JJ, Marson AG, Pearson MG. National Audit of Seizure management in Hospitals (NASH): results of the national audit of adult epilepsy in the UK. BMJ Open. 2015;5(3).
5. Hiller M, Great Britain: Parliament: House of Commons: Committee of Public Accounts. HC 502 - Services to People with Neurological Conditions: Progress Review: Stationery Office; 2016.
6. Ridsdale L, Virdi C, Noble A, Morgan M. Explanations given by people with epilepsy for using emergency medical services: A qualitative study. Epilepsy Behav. 2012;25(4):529-33.
7. Dickson JM, Dudhill H, Shewan J, Mason S, Grünewald RA, Reuber M. Cross-sectional study of the hospital management of adult patients with a suspected seizure (EPIC2). BMJ Open. 2017;7(7).
8. Reuber M, Hattingh L, Goulding PJ. Epileptological emergencies in Accident and Emergency: a survey at St James's University Hospital, Leeds. Seizure. 2000;9(3):216-20.
9. Girot M, Hubert H, Richard F, Chochoi M, Deplanque D, Derambure P, et al. Use of emergency departments by known epileptic patients: An underestimated problem? Epilepsy Research. 2015;113(Supplement C):1-4.
10. National Institute for Health and Care Excellence. Epilepsies: diagnosis and management. NICE Guideline [CG137]. 2016.
11. Luciano AL, Shorvon SD. Results of treatment changes in patients with apparently drug-resistant chronic epilepsy. Annals of Neurology. 2007;62(4):375-81.
12. Dubuc N, Bonin L, Tourigny A, Mathieu L, Couturier Y, Tousignant M, et al. Development of integrated care pathways: toward a care management system to meet the needs of frail and disabled community-dwelling older people. International Journal of Integrated Care. 2013;13:e017.
13. Iyer PM, McNamara PH, Fitzgerald M, Smyth L, Dardis C, Jawad T, et al. A Seizure Care Pathway in the Emergency Department: Preliminary Quality and Safety Improvements. Epilepsy Research and Treatment. 2012.
14. Crawford MJ, Rutter D, Manley C, Weaver T, Bhui K, Fulop N, et al. Systematic review of involving patients in the planning and development of health care. BMJ. 2002;325(7375):1263.
15. Foot C, Gilbert H, Dunn P, Jabbal J, Seale B, Goodrich J, et al. People in control of their own health and care. The state of involvement2014 29/03/2016. Available from: https://www.kingsfund.org.uk/sites/files/kf/field/field_publication_file/people-in-control-of-their-own-health-and-care-the-state-of-involvement-november-2014.pdf.
16. Palinkas LA, Horwitz SM, Green CA, Wisdom JP, Duan N, Hoagwood K. Purposeful sampling for qualitative data collection and analysis in mixed method implementation research. Administration and policy in mental health. 2015;42(5):533-44.
17. Patton MQ. Qualitative research and evaluation methods. . Thousand Oaks, CA: Sage Publications; 2002.
18. Manzano A. The craft of interviewing in realist evaluation. Evaluation. 2016;22(3):342-60.
19. The Co-ordination Centre. NHS Emergency Department Questionnaire The Co-ordination Centre: Care Quality Commission; 2016 [cited 2017 12th September]. Available from: http://www.nhssurveys.org/Filestore/AE16/AE16_Questionnaire_v1.pdf.
20. King N. 'Doing template analysis'. In: Symon G, Cassell C, editors. Qualitative Organisational Research: Core Methods and Current Challenges. London: SAGE; 2012. p. 426-50.
21. QSR. NVivo qualitative data analysis Software. QSR International Pty Ltd.; 2012.
22. Brooks J, McCluskey S, Turley E, King N. The Utility of Template Analysis in Qualitative Psychology Research. Qualitative Research in Psychology. 2015;12(2):202-22.
23. Elo S, Kyngas H. The qualitative content analysis process. J Adv Nurs. 2008;62.

For peer review only - http://bmjopen.bmj.com/site/about/guidelines.xhtml

24. Armstrong D, Gosling A, Weinman J, Marteau T. The Place of Inter-Rater Reliability in Qualitative Research: An Empirical Study. Sociology. 1997;31(3):597-606.
25. Ritchie J, Spencer L. Qualitative data analysis in applied policy research. In: Bryman A, Burgess R, editors. Analyzing Qualitative Data. London: Routledge; 1994.
26. Pope C, Ziebland S, Mays N. Analysing qualitative data. BMJ : British Medical Journal. 2000;320(7227):114-6.
27. Anderson RT, Camacho FT, Balkrishnan R. Willing to wait?: The influence of patient wait time on satisfaction with primary care. BMC health services research. 2007;7:31-.
28. Topacoglu H, Karcioglu O, Ozucelik N, Ozsarac M, Degerli V, Sarikaya S, et al. Analysis of factors affecting satisfaction in the emergency department: A survey of 1019 patients. Advances in Therapy. 2004;21(6):380-8.
29. Bleustein C, Rothschild DB, Valen A, Valatis E, Schweitzer L, Jones R. Wait times, patient satisfaction scores, and the perception of care. The American journal of managed care. 2014;20(5):393-400.
30. National Audit Office. Emergency admissions to hospital: managing the demand. London: The Stationery Office; 2013.
31. Noble AJ, Snape D, Goodacre S, Jackson M, Sherratt FC, Pearson M, et al. Qualitative study of paramedics' experiences of managing seizures: a national perspective from England. BMJ Open. 2016;6(11).
32. Purdy S. Avoiding hospital admissions. What does the research say? : The Kings Fund; 2010.
33. Leenen LAM, Wijnen BFM, van Haastregt JCM, de Kinderen RJA, Evers SMAA, Majoie MHJM, et al. Process evaluation of a multi-component self-management intervention for adults with epilepsy (ZMILE study). Epilepsy Behav. 2017;73(Supplement C):64-70.
34. NHS Institute for Innovation and Improvement. The Productive Ward: Releasing time to care. Learning and Impact Review. Executive Summary. Coventry: NHS Institute for Innovation and Improvement; 2010.
35. Wilson G. Implementation of releasing time to care – the productive ward. Journal of Nursing Management. 2009;17(5):647-54.
36. White M. How effective is the Productive Ward? Nursing Times. 2015;111(8):22-5.
37. Hesselink G, Flink M, Olsson M, Barach P, Dudzik-Urbaniak E, Orrego C, et al. Are patients discharged with care? A qualitative study of perceptions and experiences of patients, family members and care providers. BMJ Quality & Safety. 2012;21(Suppl 1):i39-i49.
38. Thapar AK, Stott NC, Richens A, Kerr M. Attitudes of GPs to the care of people with epilepsy. Fam Pract. 1998;15.
39. Thapar AK, Roland MO. General practitioner attitudes to the care of people with epilepsy: an examination of clustering within practices and prediction of patient-rated quality of care. BMC Fam Pract. 2005;6(1):9.
40. Freeman G, Hjortdahl P. What future for continuity of care in general practice? BMJ. 1997;314.
41. Freeman G, Hughes J. Continuity of care and patient experience2011 08/03/2017.
42. Hosking PG, Duncan JS, Sander JMW. The epilepsy nurse specialist at a tertiary care hospital—improving the interface between primary and tertiary care. Seizure. 2002;11(8):494-9.
43. Goodwin M, Higgins S, Lanfear JH, Lewis S, Winterbottom J. The role of the clinical nurse specialist in epilepsy: A national survey. Seizure. 2004;13(2):87-94.
44. NHS England. New Care Models: Vanguards- developing a blueprint for the future of NHS and care services London: NHS England; 2016.
45. Noble AJ, Goldstein LH, Seed P, Glucksman E, Ridsdale L. Characteristics of people with epilepsy who attend emergency departments: Prospective study of metropolitan hospital attendees. Epilepsia. 2012;53(10):1820-8.

For peer review only - http://bmjopen.bmj.com/site/about/guidelines.xhtml

46. Allard J, Shankar R, Henley W, Brown A, McLean B, Jadav M, et al. Frequency and factors associated with emergency department attendance for people with epilepsy in a rural UK population. Epilepsy Behav. 2017;68(Supplement C):192-5.
47. Seddon N. Quite like heaven? Options for the NHS in a consumer age. London: Civitas; 2007.
48. Department of Health. The NHS Improvement Plan- Putting People at the Heart of Public Services. London: The Stationery Office; 2004.
49. Williams J, Petrov G, Kennedy U, Halpenny J, Doherty CP. Moving evidence based guidelines for seizures into practice in the emergency department: What's stopping us? Epilepsy Behav. 2017;72:72-7.
50. Department of Health. The NHS Constitution. London: Williams Lea; 2015.

For peer review only

For peer review only - http://bmjopen.bmj.com/site/about/guidelines.xhtml

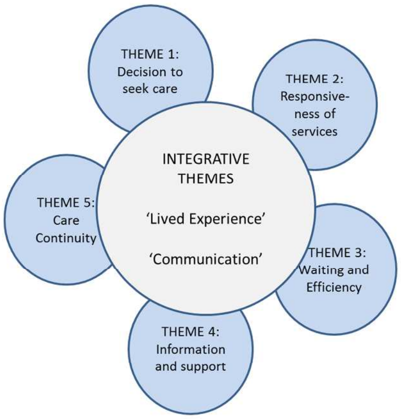

Fig.1 Schematic diagram demonstrating the five main themes identified in the study, surrounding the two central and integrative themes of 'lived experience' and 'communication'.

338x190mm (300 x 300 DPI)

SRQR Checklist

No.	Topic	Item	
	**Title and abstract**		
S1	Title	Concise description of the nature and topic of the study Identifying the study as qualitative or indicating the approach (e.g., ethnography, grounded theory) or data collection methods (e.g., interview, focus group) is recommended	S1- Page 2
S2	Abstract	Summary of key elements of the study using the abstract format of the intended publication; typically includes background, purpose, methods, results, and conclusions	S2- Page 2
	**Introduction**		
S3	Problem formulation	Description and significance of the problem/phenomenon studied; review of relevant theory and empirical work; problem statement	S3- Pages 3/4
S4	Purpose or research question	Purpose of the study and specific objectives or questions	S4- Page 6
	**Methods**		
S5	Qualitative approach and research paradigm	Qualitative approach (e.g., ethnography, grounded theory, case study, phenomenology, narrative research) and guiding theory if appropriate; identifying the research paradigm (e.g., postpositivist, constructivist/ interpretivist) is also recommended; rationale[b]	S5- Page 2
S6	Researcher characteristics and reflexivity	Researchers' characteristics that may influence the research, including personal attributes, qualifications/experience, relationship with participants, assumptions, and/or presuppositions; potential or actual interaction between researchers' characteristics and the research questions, approach, methods, results, and/or transferability	S6- Page 6
S7	Context	Setting/site and salient contextual factors; rationale[b]	S7-Page5
S8	Sampling strategy	How and why research participants, documents, or events were selected; criteria for deciding when no further sampling was necessary (e.g., sampling saturation); rationale[b]	S8- Pages 8/9
S9	Ethical issues pertaining to human subjects	Documentation of approval by an appropriate ethics review board and participant consent, or explanation for lack thereof; other confidentiality and data security issues	S9- Page 7
S10	Data collection methods	Types of data collected; details of data collection procedures including (as appropriate) start and stop dates of data collection and analysis, iterative process, triangulation of sources/methods, and modification of procedures in response to evolving study findings; rationale[b]	S10- Pages 6, 8/9
S11	Data collection instruments and technologies	Description of instruments (e.g., interview guides, questionnaires) and devices (e.g., audio recorders) used for data collection; if/how the instrument(s) changed over the course of the study	S11- Page 7
S12	Units of study	Number and relevant characteristics of participants, documents, or events included in the study; level of participation (could be reported in results)	S12- Pages 8/9
S13	Data processing	Methods for processing data prior to and during analysis, including transcription, data entry, data management and security, verification of data integrity, data coding, and anonymization/deidentification of excerpts	S13- Pages 7/8
S14	Data analysis	Process by which inferences, themes, etc., were identified and developed, including the researchers involved in data analysis; usually references a specific paradigm or approach; rationale [b]	S14- Page 8
S15	Techniques to enhance trustworthiness	Techniques to enhance trustworthiness and credibility of data analysis (e.g., member checking, audit trail, triangulation); rationale[b]	S15- Page 8
	**Results/findings**		
S16	Synthesis and interpretation	Main findings (e.g., interpretations, inferences, and themes); might include development of a theory or model, or integration with prior research or theory	S16- Pages 10-18
S17	Links to empirical data	Evidence (e.g., quotes, field notes, text excerpts, photographs) to substantiate analytic findings	S17- Pages 10-18
	**Discussion**		
S18	Integration with prior work, implications, transferability, and contribution(s) to the field	Short summary of main findings; explanation of how findings and conclusions connect to, support, elaborate on, or challenge conclusions of earlier scholarship; discussion of scope of application/ generalizability; identification of unique contribution(s) to scholarship in a discipline or field	S18- Pages 18-21
S19	Limitations	Trustworthiness and limitations of findings	S19- Pages 21/22
	**Other**		
S20	Conflicts of interest	Potential sources of influence or perceived influence on study conduct and conclusions; how these were managed	S20- Page 23
S21	Funding	Sources of funding and other support; role of funders in data collection, interpretation, and reporting	S21- Page23

[a]The authors created the SRQR by searching the literature to identify guidelines, reporting standards, and critical appraisal criteria for qualitative research; reviewing the reference lists of retrieved sources; and contacting experts to gain feedback. The SRQR aims to improve the transparency of all aspects of qualitative research by providing clear standards for reporting qualitative research.

[b]The rationale should briefly discuss the justification for choosing that theory, approach, method, or technique rather than other options available, the assumptions and limitations implicit in those choices, and how those choices influence study conclusions and transferability. As appropriate, the rationale for several items might be discussed together.

ACADEMIC MEDICINE

BMJ Open

Perceptions of emergency care using a seizure care pathway for patients presenting to emergency departments in the North West of England following a seizure; a qualitative study.

Journal:	*BMJ Open*
Manuscript ID	bmjopen-2017-021246.R2
Article Type:	Research
Date Submitted by the Author:	01-Jul-2018
Complete List of Authors:	Male, Leanne; University of Liverpool, Department of Neuroscience<br>Noble, Adam; University of Liverpool, Department of Psychological Sciences<br>Snape, Darlene; University of Liverpool, Department of Psychological Sciences<br>Dixon, Peter; University of Liverpool, Department of Biostatistics<br>Marson, Tony; University of Liverpool, Institute of Translational Medicine; The Walton Centre NHS Foundation Trust
**Primary Subject Heading**:	Health services research
Secondary Subject Heading:	Emergency medicine, Neurology, Qualitative research
Keywords:	ACCIDENT & EMERGENCY MEDICINE, Organisation of health services < HEALTH SERVICES ADMINISTRATION & MANAGEMENT, Quality in health care < HEALTH SERVICES ADMINISTRATION & MANAGEMENT, Epilepsy < NEUROLOGY, QUALITATIVE RESEARCH

SCHOLARONE™
Manuscripts

For peer review only - http://bmjopen.bmj.com/site/about/guidelines.xhtml

V11 25/06/2018

Perceptions of emergency care using a seizure care pathway for patients presenting to emergency departments in the North West of England following a seizure; a qualitative study.

Authors

Leanne Male *, Dr. Adam Noble *, Dr. Darlene Snape*, Dr. Peter Dixon*, Prof. Tony Marson *,¶

* University of Liverpool, UK ¶ The Walton Centre NHS Foundation Trust, Liverpool, UK

Corresponding Author

Address: Room 2.29, Clinical Sciences Centre, Aintree University Hospital, Lower Lane, Fazakerley, L9 7LJ. **Email:** hllmale@student.liv.ac.uk

Co-Authors

Dr. Adam Noble, University of Liverpool, Department of Psychological Sciences, Liverpool, UK

Dr. Darlene Snape, University of Liverpool, Department of Psychological Sciences, Liverpool, UK

Dr. Peter Dixon, University of Liverpool, Department of Biostatistics, Liverpool, UK

Prof. Tony Marson, University of Liverpool, Institute of Translational Medicine, Liverpool, UK The Walton Centre NHS Foundation Trust, Liverpool, UK.

Word Count- 6009

Keywords

Emergency medical services, seizures, epilepsy, qualitative research, process evaluation

For peer review only

Perceptions of emergency care using a seizure care pathway for patients presenting to emergency departments following a seizure; a qualitative study.

Abstract

Objectives To establish the appropriateness of a previously developed seizure care pathway by exploring to what extent patients valued the intervention and perceived it as being helpful or not.

Design Qualitative descriptive study, using semi-structured, in-depth interviews and thematic template analysis, theoretically informed by critical realism.

Setting In North-West England, a seizure care pathway has been developed in collaboration with a specialist neurology hospital, to support clinical management of seizure patients on initial presentation to the emergency department (ED), as well as access to follow-up services on discharge, with the aim of improving patient experience. Three National Health Service EDs and a specialist neurology hospital provided the setting for participant recruitment to this study.

Participants 181 patients fulfilled the inclusion criterion with 27 participants taking part following their experience of an ED attendance and outpatient follow-up appointment after a seizure.

Results Five main themes emerged from the data; decision to seek care, responsiveness of services, waiting and efficiency, information and support, and care continuity. Two integrative themes spanned the whole study; lived experience and communication. This paper reports on two of the main themes; care continuity and waiting and efficiency. The average time between ED presentation and interview completion was 100 days.

Conclusions Implementation of a care pathway is a complex intervention, requiring long-term follow-up to assess its integration into practice and effectiveness in service improvement. The seizure care pathway has the potential to enhance the care of seizure patients in the ED and at follow-up by improving continuity and management of care. The study demonstrates good aspects of the seizure care pathway as observed by patients, but also recognises shortcomings within current service provision and questions what the NHS should and should not be delivering. Our study suggests various ways to enhance the pathway at service-level to potentially drive improved patient experience.

For peer review only - http://bmjopen.bmj.com/site/about/guidelines.xhtml

Strengths and limitations of this study

- This is the first in-depth study exploring patient experience of an ED seizure care pathway in the UK.
- The study allows the voices of both patients with known epilepsy and those experiencing their first seizure to be heard, as well as their carers/relatives, allowing for data to be collected from multiple viewpoints.
- Despite presenting at different NHS trusts, participants often reported similar experiences; issues may be more widespread and it would be of interest to conduct further studies nationally.
- The study did not capture the perspectives of staff regarding the delivery of the care pathway in practice, which may give further insight into the appropriateness and effectiveness of its use.
- It remains to be established whether addressing the issues raised by participants about their experience of the pathway would translate into improved care quality and patient experience.

For peer review only - http://bmjopen.bmj.com/site/about/guidelines.xhtml

Introduction

The NHS, like other health systems around the world, is operating within a context of rising demand, slow growth in funding and increasing operating costs. As a consequence, NHS providers ended 2015/16 with a funding deficit of £2.45 billion [1]. The Five Year Forward View challenges the NHS to make unprecedented savings [2]. One way to achieve this is to reduce avoidable use of expensive emergency hospital services.

In the UK, seizures account for 3.3% of all calls to the emergency ambulance service [3], ~60,000 seizure-related ED attendances and ~40,000 hospital admissions each year, at a cost to the NHS in 2015/16 of ~£70 million [4-6]. Emergency care for seizures can be appropriate, important, and even life-saving. Despite this, evidence suggests that seizure patients' attendance at the emergency department (ED) is, more often than not, clinically unnecessary [7-9].

Frequently, seizure-related ED visits are though expressions of sub-optimal ambulatory care. The National Audit of Seizure management in Hospitals (NASH) found that most of those presenting to the ED with known epilepsy (~65%) had not been seen an epilepsy specialist within the past 12 months, but were in need of such support. They were often treated using sub-optimal, outdated drug treatment regimens and so at risk of avoidable seizures and/ or adverse events [4]. NICE (2016) recommend when seizures are not controlled or treatment fails, the patient should be referred to a specialist for assessment [10]. Evidence suggests such input could help improve patient outcomes [11]. ED visits do not, however, currently lead to most attendees receiving such specialist support [4].

Part of the solution might lie with the introduction of a clinical care pathway within acute hospitals. Care pathways have previously been used effectively to improve care coordination in other conditions, however there is limited evidence of their use in seizure care at present [12]. Importantly, a recent study in the Republic of Ireland saw the implementation of a pathway specifically for seizures. It was designed to improve clinical management, reduce unnecessary admissions, minimise length of stay following ED attendance and improve follow-up services through a rapid access clinic [13]. Initial evidence suggests the pathway has utility in reducing length of stay for patients and time until the patient is followed-up by outpatient services [13].

For peer review only - http://bmjopen.bmj.com/site/about/guidelines.xhtml

Ensuring equitable access to specialist neurological care in the UK has long been challenging. The above evidence suggests instituting a more proactive and better coordinated model of care may hold promise in driving up care quality while at the same time reducing emergency service use for seizures. Within the Cheshire and Mersey region of England, a seizure care pathway was therefore developed. Seizure services in the area had been identified as a key target for improvement and better co-ordination [4]. The Cheshire and Merseyside Strategic Clinical Network (SCN) co-ordinated the development of the pathway for adults presenting to emergency services with seizures in collaboration with stakeholders (neurologists, general practitioners, patient and public representatives and commissioners).

The pathway constitutes a paper proforma which provides clinical advice on patient management and investigation, and instructions as to next steps and how to access the neurology/seizure service. Patients should be made aware of the seizure care pathway, offered an advice leaflet, be informed that they will be referred to the Walton Centre NHS Foundation Trust which is the region's tertiary neuroscience hospital (and to expect to hear about an appointment in due course. Following patient ED assessment, the proforma is faxed within 24 hours to the Walton Centre appointments office for processing. Patients will then be contacted by telephone to organise a neurology out-patient appointment in a time appropriate manner; ideally within 2 weeks of their ED attendance, as per NICE guidance [10].

The Care After Presenting with Seizures (CAPS) study, funded by the National Institute of Health Research (CLAHRC NWC), was set up to evaluate the pathway's effect. CAPS is a non-randomised trial comparing the effect of the pathway to usual care alone in reducing the number of emergency visits made by patients with seizures attending three local hospital EDs .

Government policy has, for a number of years, promoted the encouragement of patients to contribute to the planning and development of health care services as a means to providing them with a stronger voice in relation to decisions around their own health and care and to facilitate service development that reflects service-user needs and preferences [2, 14]. The Five Year Forward View outlines the need for change in the relationship between people and the NHS; empowering patients to be actively involved in their own care, including working collaboratively on the development of health service delivery plans [2]. Such active

For peer review only - http://bmjopen.bmj.com/site/about/guidelines.xhtml

patient involvement has been shown to improve outcomes and experiences for patients [15]. Whilst the seizure care pathway has been designed with patient involvement, it remains important to continue this involvement following implementation of the pathway in clinical practice, to help ensure it meets patients' needs, as well as clinical requirements.

This paper presents the findings of a qualitative study of patient experiences' of the seizure care pathway. Specifically, it aimed to explore: 1) whether the pathway was valued by patients and, if so, why?; and, (2) what aspects of the intervention were perceived by patients as being helpful or unhelpful and why.

Methods

Study Design and Setting

This work is a qualitative study conducted with semi-structured patient interviews. We were interested in a descriptive evaluation of the seizure care pathway based on participants' understanding, perceptions and experiences, both in relation to their ED care following a seizure presentation and the subsequent follow-up service offered.

Participants

Table 1 outlines the inclusion/exclusion criteria for the study. Screening was undertaken by LM, a PhD student with a nursing background.

Table 1
Participant inclusion/exclusion criteria for study

Inclusion Criteria	Exclusion Criteria
• Aged 16 years or older.<br>• Presented in ED following a suspected seizure, regardless of whether they had a previous clinical diagnosis of epilepsy.	• Provoked seizure (e.g. alcohol withdrawal).<br>• Event considered unlikely to be an seizure (e.g. syncope).<br>• Reside outside of Walton Centre's catchment area<br>• Learning disability.<br>• Not fluent in written or spoken English.

For peer review only - http://bmjopen.bmj.com/site/about/guidelines.xhtml

Participants were recruited using purposive sampling, as detailed in the results section, to allow representation of the diverse range of patients attending the ED following a seizure [16, 17].

Patient and Public Involvement

Patient involvement began at the outset during the design stages of the study; development of the topic guide and research questions. The project was supported by a local charity where public support was received and utilised in the form of recruiting pilot interviewees and support with topic guide development through discussion and sharing of ideas around patient preferences, priorities and previous experiences.

Results will be disseminated to participants through a lay summary of findings posted out to all participants following the analysis of all interviews.

Data Collection

Following ethical approval from Wales NHS Research Ethics Committee (Ref. 15/WA/0207), potentially eligible patients were contacted to discuss the study further and, if interested, a time and date for interview was arranged. Participant consent was obtained prior to commencement of the interview. Face-to-face interviews were conducted with the participant and, if requested by the participant, a carer - who had been present at the time of the ED attendance – was also invited to contribute. Interviews were audio recorded and transcribed verbatim.

The interview topic guide (Table 2) was developed through a scoping review of the relevant literature. The topic guide was piloted and iterative data analysis, researcher reflections and study team discussions were utilised to update the topic guide throughout the process of data collection [18].

Table 2
Specific topics introduced within interviews.

Figure 1. Topic guide for interviews
Can you tell me about what happened to make you attend ED?<br>Probe: experience, decision-making, transport to ED.

For peer review only - http://bmjopen.bmj.com/site/about/guidelines.xhtml

Tell me what you remember about your experience in the ED.<br>Probe: Care processes, interactions with professionals, involvement in decision-making, information and support provision.
Was the most recent experience any different to past experiences of ED?<br>Probe: similarities/differences, likes/dislikes, changes.
What does 'good care' mean to you?<br>Probe: expectations, shortfalls.<br>What sort of changes, if any, could be made to improve your care throughout different stages in the ED?
Tell me about your experience of the transition of care between ED and the Walton Centre?<br>Probe: communication, timeliness, similarities/differences to previous referrals.
Can you tell me about your experience of when you went for your outpatient clinic appointment?<br>Probe: usefulness of appointment, benefits of pathway, improvement suggestions, how well do you feel your 'crisis' was dealt with?
Discuss the usefulness of the current ED Questionnaire (Picker Institute) from patients' opinion[19].

Data analysis

Interviews were analysed thematically using the template method, informed by the work of King [20]. QSR International's NVivo V.10 software [21] was used to facilitate data management and document the analytical process. The analytic template was developed using a combination of *a priori* codes, derived from previous research around patient experience in general, and salient findings from early participant interviews. Using *a priori* codes ensured a focus on key areas potentially relevant to the study [22]. Further codes and themes were identified deductively through analysis of raw data, allowing identification of themes and patterns related to the study aims [23].

The initial template was developed by LM and another researcher (DS). Transcripts were coded separately by LM and DS before comparison was made using the coding template [24]. Transcripts were read and re-read, further amendments were made to the template until coding consensus was reached [25, 26]. Final themes were reviewed by the study team to ensure they addressed the subject matter and accounted for the data content.

For peer review only - http://bmjopen.bmj.com/site/about/guidelines.xhtml

Findings

Participant characteristics

Participants who met the inclusion criteria for CAPS, and had received a follow-up appointment following their ED visit, were invited to participate in the qualitative study. One hundred and eighty-one patients were screened for eligibility and contacted via telephone to invite them to take part in the study. Of these, 17 patients declined to participate. Reasons given for their refusal included anxiety, poor memory, and time constraints. A further 137 potential participants could not be accessed due to patients not answering telephone calls/ not replying to messages left, and incorrect patient contact details.

The overall acceptance rate was therefore 22%, with 15% of those screened eventually participating. A total of twenty seven interviews were conducted between March 2016 and May 2017. Twenty five of the interviews were conducted in the participant's own home, with the remaining two interviews, taking place at an alternative location at the request of the participant. The average time between participants' ED presentations and interviews was 100 days (SD 32.6). Demographics and clinical characteristics of the study sample are detailed in Table 3. For clarity, 'Not epileptic Seizure', includes both non-epileptic attack disorder (NEAD) as well as other presentations referred on to neurology such as syncope.

Table 3
Patient demographics and clinical characteristics, grouped according to diagnosis following neurology review.

	Known Epilepsy (n=13)	First unprovoked Seizure (n= 8)	Not epileptic Seizure (n=6)
**Age (years)**			
-Mean	50	37	32
-Range	24-78	17-64	19-45
**Gender**			
-Male	6 (46.2%)	5 (62.5%)	4 (66.7%)
-Female	7 (53.8%)	3 (37.5%)	2 (33.3%)
**Employment**			
-Full time work	3 (23.1%)	1 (12.5%)	2 (33.3%)

For peer review only - http://bmjopen.bmj.com/site/about/guidelines.xhtml

-Part time work	2 (15.4%)	1 (12.5%)	0 (0%)
-Not employed	8 (61.5%)	5 (62.5%)	3 (50%)
-Education	0 (0%)	1 (12.5%)	1 (16.7%)
**IMD Regional Decile**			
-1-3 (most deprived)	9 (69.2%)	4 (50%)	3 (50%)
-4-6	2 (15.4%)	3 (37.5%)	2 (33.3%)
-7-10 (least deprived)	2 (15.4%)	1 (12.5%)	1 (16.7%)

Identified themes

Analysis of the transcripts elicited five key themes (Figure 1). In addition, two integrative concepts: 'Lived Experience' and 'Communication' permeated participant discussions and as such were common across all themes.

The present paper focuses on findings from two of the five top level themes specifically due to the level of in depth description required to adequately explain such complex themes; namely, 'waiting and efficiency' and 'care continuity' as two of the most discussed themes within interviews Care continuity, both within the ED and across services, was highlighted as the most important to participants in relation to their overall experience of the care pathway, along with waiting, which was frequently referred to throughout interviews. Each theme/sub-theme will be discussed in turn in relation to participants' perception of the pathway as an effective intervention and examine each in relation to its perceived value to the pathway process overall.

Waiting and efficiency

Time was viewed by most participants as a factor influencing experience. Irrespective of whether the participant perceived their treatment as being fast or slow, time was continually mentioned, highlighting its importance in the process experienced by participants.

Participants' experience of waiting times varied at different points in their journey through the ED. Reference was repeatedly made to '*waiting for triage*', '*waiting for tests*' and '*waiting to see a doctor*'. When participants perceived their wait time as being '*quick'*, particularly in comparison to previous experience, they reported positive feedback on the process.

For peer review only - http://bmjopen.bmj.com/site/about/guidelines.xhtml

'there was no question of she's going to wait or whatever; come right through and I say I saw that doctor…they were absolutely brilliant I couldn't fault them at all.' (P8, Female, First unprovoked seizure)

Conversely, participants perceived 'long' waiting contributed to poor experience.

'if your waiting on one of them beds to see a doctor…it does make you feel a bit on edge…people just get upset sitting out there.' (P29, Male, Known epilepsy)

Waiting and environmental resources

Participants frequently described the physical environment of the ED as "*busy*". A commonly reported feature was lack of bed space within the department, resulting in participants being left on trollies in the ED corridor for significant periods of time. As one female explained:

'they were so backed up that we came in via ambulance and you know the way they have all the trolleys down the corridor backed up, they were that bad that the paramedics had to stay with me for 3 hours in a corridor.' (P5, Female, Known epilepsy)

Similarly, this scenario had a significant impact on another participant's ED experience, prompting his self-discharge from the service. In this instance, the participant reported being in a post-ictal state but felt he no longer particularly required treatment; a decision informed, in part, by the belief he was a burden to the paramedics who had to remain with him during his time on a trolley in the corridor. He explained:

'there was the guilt trip I suppose on the last one because I had, this was adding to the anxiety because I was stuck on a trolley with paramedics looking after me and I'm thinking they could be out saving someone else's life. So that added to the pressure if that makes sense. Erm and that's why I said look you can't wait any longer and so I signed a self-discharge form because I felt guilty.' (P32, Male, Known epilepsy)

Further interviews revealed participants' observations about the environment (often described as "*busy*" and "*noisy*") as well as their views about how it could be improved in a

For peer review only - http://bmjopen.bmj.com/site/about/guidelines.xhtml

way that was more conducive to supporting those following a seizure. Suggestions for side rooms, dimmed lighting, and a quieter area to enable patients to recover from their seizure in a calm and timely manner were articulated.

> *'…they realised then they had to put me in a dark room to take my bloods and everything and then they said we'll try and put you somewhere quiet… they moved me up to the next waiting area… again there was the television on, flashing lights and we said you know you can't do that. If you put me in a dark room where it is nice and quiet my seizures will stop and calm down but they won't listen.'* (P5, Female, Known epilepsy)

Time for care

Participants not only spoke about time in relation to waiting, but also with reference to time health care professionals (HCPs) spent engaging with them. While the majority of participants reported being satisfied with the time spent with HCPs, the visibility of staff members, and the communication they received, others spoke of negative experiences related to the limited time spent specifically with HCPs.

Participants reported that waiting for a doctor to arrive often induced fear and uncertainty; triggering worrying thoughts about what might be wrong and what would happen next. This feeling was articulated mainly by those participants experiencing a first seizure, for whom the event and ED environment was largely unfamiliar. Overall, participants commonly held the view that doctors and nurses were "*in a rush*". Such observations and experiences led to a sense of "*urgency*", felt by participants and a perceived inadequacy in support offered by HCPs.

> *'I don't know how long the doctor was with me or the nurse but it didn't seem very long at all… yes I would have liked to have known more and the amount of time I spend in there, it seemed a very little time I spent with the doctor or the nurse.'* (P12, Male, Known epilepsy)

Those with known epilepsy tended to say they were more relaxed about the visibility of HCPs, not least because they expressed being more *"in control*" of the situation. More often than not, these participants reported a more positive experience of visibility of HCPs and

For peer review only - http://bmjopen.bmj.com/site/about/guidelines.xhtml

were contented with the amount of time spent in consultation with doctors and nurses; even if this time was minimal and contact related mainly to routine basic observations.

> *'we were pretty much left to our own devices really, they checked that she was ok and then off they went. They weren't really regular check-ups because its more just waiting for her to come back round and be lucid and waiting for a doctor to turn up.'* (P6, Female, Known epilepsy, Carer)

In contrast, a female with known epilepsy expressed great concern about the lack of contact with HCP's. Her concern led to fears for safety and a sense of heightened insecurity about the level of care received. These fears occasioned a negative perception of the care pathway and general experience within the ED.

> '*there was no medical staff available because they were so busy… I felt in danger because nobody knew what they were doing.'* (P5, Female, Known epilepsy)

Conceptualisation of service provision

Despite acknowledging shortfalls in the service provided, often participants expressed understanding as to why this may be the case. Most commonly, limited staff and resources in the ED were highlighted as a potential cause of increased waiting times.

> '*it was just queue of trollies with people in it. It was 4 and a half/5 hours just sat, lying around waiting… I didn't see anyone I was just lying there.'* (P26, Male, First unprovoked seizure)

Participants also noted 'time of day' at which they attended the ED as a possible causal factor for increased wait; with certain times and days being recognised as particularly busy. These times included evenings, with particular reference to Friday and Saturday nights. At these times participants said they believed EDs are regularly frequented by individuals who are intoxicated or who have injuries associated with alcohol and illicit drug use.

For peer review only - http://bmjopen.bmj.com/site/about/guidelines.xhtml

> *'it depends on what time of day you go, it depends on how you go, get taken into A&E it affects everything you know.'* (P2, Female, First unprovoked seizure)

> *'if it was 10 o'clock on a Saturday night it would be totally different wouldn't it…'* (P8, Female, First unprovoked seizure)

Often there were negative feelings expressed about the use of EDs in relation to alcohol and drug use. In particular the view that attendance under such circumstances represented service misuse.

> *'I think they were just happy to have a call out on a Friday night that wasn't a drunk in town basically. It was just an easy job for them.'* (P6, Female, Known epilepsy)

Finally, some participants recognised the use of the triage system, and the need to prioritise care of "*other, sicker patients*" as a cause for delays in their own treatment. Participants with seizures had typically been conveyed to ED via ambulance. This was seen by one participant as a quicker way into ED than the "*normal*" route of self-referral to ED and not attending by ambulance.

> *'yes the normal way I think it is longer but with the ambulance taking you I think you get seen quicker.'* (P1, Female, Known epilepsy)

Care Continuity

Three second-order themes were defined within the main theme of 'care continuity'; perceptions of care continuity, accessibility of services responsive to need, and sense of abandonment.

Perceptions of continuity

According the pathway proforma, the patients attending doctor within the ED is responsible for informing the patient of the subsequent stages in their care pathway. This should be a timely referral for a neurology outpatient appointment. When asked about their understanding of this process, most participants acknowledged being told about the follow-up procedure, highlighting a positive example of communication within the ED.

For peer review only - http://bmjopen.bmj.com/site/about/guidelines.xhtml

In the few cases where participants were not told about the pathway, or when their waiting time for follow-up was delayed, this led to concern and anxiety, particularly among first seizure participants.

Conversely, those with known epilepsy expressed lower levels of anxiety in response to lack of advisement around follow-up procedures. Such participants often demonstrated their knowledge of the health service system; proactively accessing services they have previously used to obtain the follow-up care they wanted. This finding highlights that patients with known epilepsy, when provided with appropriate means, are able to proactively seek care to address their needs.

> *'it's like we have our own pathway that just sort of made ourselves… but that sort of works because of the fact that for her condition it's easier to manage everything ourselves so we know what's going on. As far as the only pathway is when all the information gets handed over if she's got an appointment she wouldn't know what it's for. Whereas once she's booked one directly with [epilepsy specialist nurse] she knows why she's going and what she wants to talk about.'* (P6, Female, Known epilepsy)

Epilepsy specialist nurses (ESN) were only mentioned by participants with known epilepsy. In this group participants spoke positively of the role and knowledge of nurse specialists. As one participant noted:

> *'so [epilepsy specialist nurse] is absolutely brilliant… yes she's quite on the ball.* (P6, Female, Known epilepsy)

However, those participants who were new to the service did not demonstrate any knowledge or understanding of the ESN role or related service provision.

Accessibility of services to suit need

Participants experienced numerous barriers when transitioning from emergency care to follow-up ambulatory services. Important barriers were mainly organisational whereby participants frequently recounted experience of: difficulty in negotiating the appointment system, delays in receiving referral appointments, difficulty in accessing hospital/ clinic locations and related transport problems.

Some participants noted that travelling to appointments was difficult. A combination of ill-health, inability to drive due to seizures, cost of taxis, and poor public transport connections, were noted as barriers to accessing services. This led to patients being reliant

For peer review only - http://bmjopen.bmj.com/site/about/guidelines.xhtml

on family members to support them to get to their appointments; increasing a sense of burden and dependence in patients who were otherwise independent.

Where participants were expected to attend for more than one follow-up appointment, for example where scans and further tests were required, they felt this compounded the level of inconvenience they experienced. Participants questioned the current process around 'follow-up' care provision. It was suggested, as a means to reducing the level of inconvenience experienced by patients, that appointments be combined to reduce the number of visits, or, if possible, appointments be conducted in a local district general hospital (DGH) suitable to the patient's location, thereby reducing the resources required for access.

> *'…because I can't drive now because of the epilepsy and I'm on my own with the children it's tricky, I've missed a couple of appointments at [tertiary clinic] just because it's tricky to get there. Because if I can't get anyone to give me a lift and look after the children it's hard for me to get there... it would be easier if they could come to my local doctors and do a clinic there.'* (P35, Female, Known epilepsy)

> '*I had to go back to [tertiary hospital] every day… and obviously from here it's like an hour and it was all that petrol so my dad had to take the week off work and take me back there every day before 9am which was tiring.'* (P36, Female, Known epilepsy)

Sense of abandonment

Participants reporting of follow-up care after ED attendance varied significantly, demonstrating that all patients' who had access to the pathway at ED did not necessarily receive the same benefits of the intervention post-ED discharge. Frequently, and irrespective of the level of information provided at ED discharge, this lack of continuity left patients feeling *"abandoned"*. This situation occurred predominantly at two time points; immediately after discharge from ED, and following outpatient review at neurology clinic.

After discharge from ED, participants reported expecting to receive a follow-up appointment letter from the Walton Centre. In some instances, participants spoke of experiencing significant delays in receiving this appointment, or not receiving an appointment. These experiences led participants to feel anxious, expressed as not knowing where to turn for further support. In addition, participants reported being faced with feelings of uncertainty about what to do and how long to wait before seeking further help.

For peer review only - http://bmjopen.bmj.com/site/about/guidelines.xhtml

'it was just frustrating not knowing what was going on and then the fact I had to chase it up the appointment. If I hadn't chased it up three times I'd have never got the see the neurologist… I even went to the doctors [GP] and they [GP] left it up to me to chase them up and that is definitely wrong.' (P14, Female, Not epileptic seizure)

The above quote reflects the actions of many participants within the study. Those having problems with hospital follow-up appointments often reported accessing their general practitioner (GP) for assistance. This scenario however was seen to create further frustration with participants often feeling disappointed with GP care. Not least because it was felt the GP could not deliver the level of support needed by the participant and that clinical decisions were often referred back to neurology services.

Conversely, some participants reported feeling well supported by their GP with regard to their seizures. Although it was noted that GPs were often reluctant to make decisions with regard to anti-epileptic drugs, the support and advice offered by GPs, through liaison with neurology services, had helped some participants feel aided to manage their condition without having to access emergency care.

'she's [GP] been brilliant, she's been amazing, really, really good… I can't complain and you know if there was a problem I could just speak to the GP or I could ring someone at [Walton Centre] that's not a problem. I know there's always somebody available if you've got concerns.' (P35, Female, Known epilepsy)

The second juncture when participants recounted feelings of abandonment was following their follow-up outpatient appointments. This more commonly occurred in patients who had experienced a first seizure, or a formal epilepsy diagnosis was not reached. These patients were typically discharged back to their GP after any necessary tests. When seizures continued participants were uncertain about what to do; often not seeing the GP as adequately trained to deal with such situations. This sense of abandonment was underpinned by beliefs that the service could not meet the needs of individuals. This led to frustration in relation to care, increasing anxiety and feelings of low mood within participants.

For peer review only - http://bmjopen.bmj.com/site/about/guidelines.xhtml

'I did take the letter in that the neurologist sent to me but all she [GP] kept doing was reading through it and typing what that letter says onto there's. She wasn't doing her own, I don't know, her own idea of what it could be or something I think she was just copying what the neurologist said because she didn't have… in my eyes she didn't have a clue in that doctors.' (P26, Male, First unprovoked seizure)

Participants repeatedly called for GPs to be better trained to deal with epilepsy and seizures. Participants believed this would enable GP's to provide appropriate, interim support while patients waited for outpatient appointments, or during times between outpatient appointments and following discharge from neurology. In one particular case; a participant diagnosed with non-epileptic attack disorder (NEAD), lack of knowledge and understanding of the specific condition by the GP was felt to have hindered further follow-up care in the primary care setting resulting in a significant negative impact on her quality of life.

> *'I don't think she [GP] really knows what it is to be honest with you or how it affects you…it's such a new thing NEAD that they don't know what it is. Like I said I've never heard of it…if you had a seizure you had epilepsy basically that's what people think isn't it… I think there needs a lot more training in it because the way it seems, because they say it's in your head and you get people saying well if it's in your head you can get rid of it.''* (P14, Female, Not epileptic seizure)

This sense of abandonment and not knowing where to turn in time of 'crisis', on occasion, resulted in participants reporting overuse of the ED. Returning to the ED following each seizure, although recognised as not clinically necessary, was seen by some participants as a way back into the system. As one participant explained:

> *'I mean a lot of the time obviously again they email [tertiary hospital], they email your GP to say you've been there [A&E] you know and the doctor will look at that and just go right what's going on here why has she gone to A&E again and that's when they probably bring the appointment forward but yeah it makes you more inclined definitely to go to A&E… just to get seen and not have to wait weeks on end, months on end just for an appointment that's going to take 10 minutes… at least then even if you tell them what's going on at least they know what they're doing.'* (P36, Female, First unprovoked seizure)

For peer review only - http://bmjopen.bmj.com/site/about/guidelines.xhtml

Discussion

To our knowledge, this is the first study to explore patient experiences and perceptions of an ED seizure care pathway. Despite variation in care experience throughout the process, some common themes were evident.

Waiting times and patient perceived *efficiency* within the service are fundamental elements of the care process. Wait times that were perceived to be significant contributed to poorer experiences of care. These findings are broadly consistent with other studies that demonstrate longer waits are associated with lower levels of patient satisfaction [27-29]. In the previous 15 years emergency hospital attendances have increased by 47%, resulting in increased pressures on EDs [30]. Waiting in corridors was seen as an acceptable and somewhat 'standard' procedure to participants who had previous knowledge or experience within the ED.

Some participants described feelings of self-perceived burden as a result of their seizure attendance. This finding suggests that some patients in a post-ictal state may feel that their attendance at the ED is unnecessary and that they recognise they didn't actually require the service. This is supported by a recent study conducted in a large hospital trust which concluded that the majority of people who have had seizures do not require the facilities of the ED for treatment [7].

There is a need to realign our pre-hospital emergency care system to help reduce avoidable hospital attendances and admissions. Reducing the number of patients unnecessarily conveyed to hospital via ambulance through better training of ambulance staff may be a way to reduce ED burden [31]. Interventions to support self-management in patients have the potential to reduce hospital attendances, allowing patients to feel empowered in decision-making surrounding their own health care needs [32, 33].

Making time for care

Highlighted as key in this study is HCPs having the *time for care.* Time spent directly with patients has been a subject on the NHS agenda in recent years, with implementation of the NHS Institute for Innovation and Improvement's *Releasing Time to Care- Productive Ward* initiative in 2005, designed to improve patient satisfaction by increasing direct patient care

For peer review only - http://bmjopen.bmj.com/site/about/guidelines.xhtml

by staff [34]. Positive results were seen as a consequence of the initiative, not only in improvements in patient experience, but also in clinical and safety outcomes [35, 36]. In our study, there were mixed experiences with regard to time spent directly with HCPs, with the majority reporting a positive experience, and few reporting negatively about their level of contact time. There is little evidence that the *Productive Ward* initiative [35] has been utilised within emergency medicine and our findings suggest that this could be a useful tool to help improve patient experience.

Discharge support and ongoing care

Poor care co-ordination resulted in the majority of participants reporting a *sense of abandonment* at some point in their journey along the care pathway. This included feeling unprepared to be discharged, lack of help and support from GPs, and poor administrative communication with regard to follow-up appointments; all of which frequently left patients feeling anxious and helpless in their ongoing ambulatory care. This finding concurs with findings from the wider literature, with Hesselink et al. reported ED patients in general can feel unprepared at time of discharge [37]. In our study, this finding demonstrates a shortfall in the effectiveness of the pathway process; highlighting potential for improvement, to better support patients on discharge from the ED and their ongoing ambulatory care support.

A large proportion of participants felt the GP's support in their follow-up care to be deficient for their needs. Some GPs do not feel confident in dealing with epilepsy and making medical decisions resulting in treatment delays [38, 39]. In these cases, as in our own study, patients have reported feeling frustrated and unhappy with care. Several suggestions to improve this aspect of care can be drawn from these findings. GPs have an integral role in care continuity of patients with long term conditions [40, 41]. Improvement in training of GPs in seizure management and epilepsy medications could reduce treatment delays and further ED attendances. Furthermore, GPs could take on added responsibility of providing a 'safety net' to try to help when communications break down between the ED and neurology services.

Further to the role of the GP, the ENS role is also designed to improve continuity and accessibility to neurology services [42, 43]. The Walton Centre has been named an NHS Vanguard under the New Care Models scheme which means they have national support to

For peer review only - http://bmjopen.bmj.com/site/about/guidelines.xhtml

pioneer new ways of working that benefits patients [44]. As part of this scheme, 'integrated neurology nurses' have recently been employed to bridge the gap between the centre and the community, aim to provide a better link between the hospital, the GP, and community multi-disciplinary teams.

Our findings support the suggestion of incorporating ongoing ambulatory care services, both from GPs and nurse specialists, within the care pathway. This would likely promote care quality across the continuum of the patient journey. Patients are faced with making difficult decisions when accessing care. Such decisions are based on their own understanding about what will be of most benefit and what will improve their care. Where patients feel at a loss regarding what to do next, a return to ED to prompt continuation of care has been reported as an option for some patients. There have been various studies around reasons for re-attendance [45, 46], but there is little prior evidence showing that poor follow-up care results directly in re-attendance to the ED. However, this study demonstrates that lack of adequate follow-up and *sense of abandonment,* can result in some patients' re-attending ED as a means to re-accessing the neurology system.

This present study also demonstrates that the entire seizure care pathway process needs addressing to ensure smoother patient transition and to allow patients to be better supported in their ongoing care. We recognise both areas of good practice and areas where patients' suggest improvements. These improvements, however large or small, need to be achievable in the context of the NHS. Raised patient expectations, medical and technological advances, and an increased scope in what can be treated [47] , alongside a call to "*reshape the health services around the needs and aspirations of patients*" [48], has somewhat changed patient perception of what the NHS was originally set up to deliver in relation to demands now being placed on an already struggling service. The capacity of NHS service delivery needs to be taken into consideration when reviewing suggestions made for service improvement in order to make changes that will benefit patients and staff, as well as being both clinically and financially achievable.

Strengths and limitations

Previous work in the field of seizure pathway evaluation did not qualitatively explore patient experience [13]. Our findings thus provide a more nuanced exploration of the pathway intervention, albeit sensitive to the context in which it is being delivered. Interviews were

For peer review only

conducted in the patient's location of choice. This not only advantaged recruitment as the location was more convenient for participants but also facilitated building rapport, as a non-clinical, more relaxed environment potentially encouraged participants to talk openly about their experiences. The Walton Centre catchment area is drawn from Merseyside, Cheshire, Lancashire, Greater Manchester, the Isle of Man and North Wales. The patient demographics are representative of the ED catchment area populations, where we would expect to see higher levels of unemployment and participants more likely to be living in the more deprived areas of the city.

Participants were recruited from three NHS study sites within NW England; all had a specific research nurse responsible for embedding the care pathway and follow-up procedure. This could potentially be a limitation, as the issues identified are specific to a nurse-led care pathway. In addition, this is a local initiative and as such findings from the current study may not be generalizable to other settings/ geographical locations.

Added to this, participant uptake was low (i.e., 22%). This figure may reflect a number of challenges associated with the conduct of this study including, for example, the chosen recruitment strategy, the difficulty in contacting patients by telephone and, the nature of epilepsy as an illness. It is important to also acknowledge there is an inherent bias with studies of this nature; those opting to participate may have strong positive or negative feelings about the service which could potentially bias the results.

The average time latency of 100 days from ED attendance to interview may also have influenced the amount of information participants could remember about their experience. While this could not be avoided as participants had to have had their outpatient appointment before being interviewed, therefore experiencing the whole pathway, it should be recognised as a potential limitation to the findings. .

This study did not capture perspectives of staff working within the field of the seizure care pathway. Previous quantitative work has though been conducted around staff perceptions of the barriers to implementation of a care pathway for seizure patients in Ireland [49] We recognise this as an important area likely to provide further insight around the care pathway implementation process in our study and are currently conducting a sub-study to qualitatively explore the views of these individuals.

For peer review only - http://bmjopen.bmj.com/site/about/guidelines.xhtml

Conclusions

Clinical, professional and organisational barriers all have impact on the delivery of complex interventions such as that of the seizure care pathway. That said, patients rely on such interventions, along with the support of HCPs', to support their transition from ED to ambulatory care. Although spanning a broad spectrum, there are various service-focused changes that have been suggested and discussed through our findings which may help to improve experience in this patient group. Refining interventions to support self-management, improved training for ambulance staff, and more efficient use of current services such as epilepsy specialist nurses and the 'integrated neurology nurses' could all prove useful in improving experience. It is crucial to the health service to take into consideration the suggestions made by patients in order to drive forward care based on what patients want. However, recognising the scope of the NHS, its values, and the current financial challenges, it is important to recognise service improvements that will have the most impact. This will not only drive quality of care for service users but also potentially support the NHS in reducing its current financial deficits [50].

Figure Legend

Fig.1 Schematic diagram demonstrating the five main themes identified in the study, surrounding the two central and integrative themes of 'lived experience' and 'communication'.

Acknowledgements The authors would like to thank the four NHS hospital trusts for their support with recruiting participants, patient advisers who supported the development of the study and those patients who gave their time to take part.

Contributors TM, PD, and AN conceived the initial idea of the study, designed the trial, and obtained research funding. LM recruited, carried out, and analysed the interviews, with input from DS. LM drafted the manuscript and PD, AN, DS and TM made contributions to its development. All authors reviewed and approved the final manuscript.

Funding This work was supported by the National Institute of Health Research (CLAHRC NWC). The views expressed are those of the author(s) and not necessarily those of the NHS, the NIHR or the Department of Health.

Competing interests None declared.

For peer review only - http://bmjopen.bmj.com/site/about/guidelines.xhtml

Ethics approval Ethical approvals were obtained from the research sponsor (University of Liverpool - protocol number: UoL001140) and from Wales Research Ethics Committee 7, (reference number: 15/WA/0207). Approval from HRA and the Research and Development department of each of the participating NHS hospital trusts (IRAS ID 173222) was also sought prior to commencing the study.

Data sharing statement No additional data are available.

References

1. House of Commons Committee of Public Accounts. Financial sustainability of the NHS: Forty-third Report of Session 2016-17 2017.
2. NHS England. NHS Five Year Forward View. London: NHS England; 2014.
3. Dickson JM, Taylor LH, Shewan J, Baldwin T, Grünewald RA, Reuber M. Cross-sectional study of the prehospital management of adult patients with a suspected seizure (EPIC1). BMJ Open. 2016;6(2).
4. Dixon PA, Kirkham JJ, Marson AG, Pearson MG. National Audit of Seizure management in Hospitals (NASH): results of the national audit of adult epilepsy in the UK. BMJ Open. 2015;5(3).
5. Hiller M, Great Britain: Parliament: House of Commons: Committee of Public Accounts. HC 502 - Services to People with Neurological Conditions: Progress Review: Stationery Office; 2016.
6. Ridsdale L, Virdi C, Noble A, Morgan M. Explanations given by people with epilepsy for using emergency medical services: A qualitative study. Epilepsy Behav. 2012;25(4):529-33.
7. Dickson JM, Dudhill H, Shewan J, Mason S, Grünewald RA, Reuber M. Cross-sectional study of the hospital management of adult patients with a suspected seizure (EPIC2). BMJ Open. 2017;7(7).
8. Reuber M, Hattingh L, Goulding PJ. Epileptological emergencies in Accident and Emergency: a survey at St James's University Hospital, Leeds. Seizure. 2000;9(3):216-20.
9. Girot M, Hubert H, Richard F, Chochoi M, Deplanque D, Derambure P, et al. Use of emergency departments by known epileptic patients: An underestimated problem? Epilepsy Research. 2015;113(Supplement C):1-4.
10. National Institute for Health and Care Excellence. Epilepsies: diagnosis and management. NICE Guideline [CG137]. 2016.
11. Luciano AL, Shorvon SD. Results of treatment changes in patients with apparently drug-resistant chronic epilepsy. Annals of Neurology. 2007;62(4):375-81.
12. Dubuc N, Bonin L, Tourigny A, Mathieu L, Couturier Y, Tousignant M, et al. Development of integrated care pathways: toward a care management system to meet the needs of frail and disabled community-dwelling older people. International Journal of Integrated Care. 2013;13:e017.
13. Iyer PM, McNamara PH, Fitzgerald M, Smyth L, Dardis C, Jawad T, et al. A Seizure Care Pathway in the Emergency Department: Preliminary Quality and Safety Improvements. Epilepsy Research and Treatment. 2012.
14. Crawford MJ, Rutter D, Manley C, Weaver T, Bhui K, Fulop N, et al. Systematic review of involving patients in the planning and development of health care. BMJ. 2002;325(7375):1263.
15. Foot C, Gilbert H, Dunn P, Jabbal J, Seale B, Goodrich J, et al. People in control of their own health and care. The state of involvement2014 29/03/2016. Available from: https://www.kingsfund.org.uk/sites/files/kf/field/field_publication_file/people-in-control-of-their-own-health-and-care-the-state-of-involvement-november-2014.pdf.

For peer review only - http://bmjopen.bmj.com/site/about/guidelines.xhtml

16. Palinkas LA, Horwitz SM, Green CA, Wisdom JP, Duan N, Hoagwood K. Purposeful sampling for qualitative data collection and analysis in mixed method implementation research. Administration and policy in mental health. 2015;42(5):533-44.
17. Patton MQ. Qualitative research and evaluation methods. . Thousand Oaks, CA: Sage Publications; 2002.
18. Manzano A. The craft of interviewing in realist evaluation. Evaluation. 2016;22(3):342-60.
19. The Co-ordination Centre. NHS Emergency Department Questionnaire The Co-ordination Centre: Care Quality Commission; 2016 [cited 2017 12th September]. Available from: http://www.nhssurveys.org/Filestore/AE16/AE16_Questionnaire_v1.pdf.
20. King N. 'Doing template analysis'. In: Symon G, Cassell C, editors. Qualitative Organisational Research: Core Methods and Current Challenges. London: SAGE; 2012. p. 426-50.
21. QSR. NVivo qualitative data analysis Software. QSR International Pty Ltd.; 2012.
22. Brooks J, McCluskey S, Turley E, King N. The Utility of Template Analysis in Qualitative Psychology Research. Qualitative Research in Psychology. 2015;12(2):202-22.
23. Elo S, Kyngas H. The qualitative content analysis process. J Adv Nurs. 2008;62.
24. Armstrong D, Gosling A, Weinman J, Marteau T. The Place of Inter-Rater Reliability in Qualitative Research: An Empirical Study. Sociology. 1997;31(3):597-606.
25. Ritchie J, Spencer L. Qualitative data analysis in applied policy research. In: Bryman A, Burgess R, editors. Analyzing Qualitative Data. London: Routledge; 1994.
26. Pope C, Ziebland S, Mays N. Analysing qualitative data. BMJ : British Medical Journal. 2000;320(7227):114-6.
27. Anderson RT, Camacho FT, Balkrishnan R. Willing to wait?: The influence of patient wait time on satisfaction with primary care. BMC health services research. 2007;7:31-.
28. Topacoglu H, Karcioglu O, Ozucelik N, Ozsarac M, Degerli V, Sarikaya S, et al. Analysis of factors affecting satisfaction in the emergency department: A survey of 1019 patients. Advances in Therapy. 2004;21(6):380-8.
29. Bleustein C, Rothschild DB, Valen A, Valatis E, Schweitzer L, Jones R. Wait times, patient satisfaction scores, and the perception of care. The American journal of managed care. 2014;20(5):393-400.
30. National Audit Office. Emergency admissions to hospital: managing the demand. London: The Stationery Office; 2013.
31. Noble AJ, Snape D, Goodacre S, Jackson M, Sherratt FC, Pearson M, et al. Qualitative study of paramedics' experiences of managing seizures: a national perspective from England. BMJ Open. 2016;6(11).
32. Purdy S. Avoiding hospital admissions. What does the research say? : The Kings Fund; 2010.
33. Leenen LAM, Wijnen BFM, van Haastregt JCM, de Kinderen RJA, Evers SMAA, Majoie MHJM, et al. Process evaluation of a multi-component self-management intervention for adults with epilepsy (ZMILE study). Epilepsy Behav. 2017;73(Supplement C):64-70.
34. NHS Institute for Innovation and Improvement. The Productive Ward: Releasing time to care. Learning and Impact Review. Executive Summary. Coventry: NHS Institute for Innovation and Improvement; 2010.
35. Wilson G. Implementation of releasing time to care – the productive ward. Journal of Nursing Management. 2009;17(5):647-54.
36. White M. How effective is the Productive Ward? Nursing Times. 2015;111(8):22-5.
37. Hesselink G, Flink M, Olsson M, Barach P, Dudzik-Urbaniak E, Orrego C, et al. Are patients discharged with care? A qualitative study of perceptions and experiences of patients, family members and care providers. BMJ Quality & Safety. 2012;21(Suppl 1):i39-i49.
38. Thapar AK, Stott NC, Richens A, Kerr M. Attitudes of GPs to the care of people with epilepsy. Fam Pract. 1998;15.

For peer review only - http://bmjopen.bmj.com/site/about/guidelines.xhtml

39. Thapar AK, Roland MO. General practitioner attitudes to the care of people with epilepsy: an examination of clustering within practices and prediction of patient-rated quality of care. BMC Fam Pract. 2005;6(1):9.
40. Freeman G, Hjortdahl P. What future for continuity of care in general practice? BMJ. 1997;314.
41. Freeman G, Hughes J. Continuity of care and patient experience2011 08/03/2017.
42. Hosking PG, Duncan JS, Sander JMW. The epilepsy nurse specialist at a tertiary care hospital—improving the interface between primary and tertiary care. Seizure. 2002;11(8):494-9.
43. Goodwin M, Higgins S, Lanfear JH, Lewis S, Winterbottom J. The role of the clinical nurse specialist in epilepsy: A national survey. Seizure. 2004;13(2):87-94.
44. NHS England. New Care Models: Vanguards- developing a blueprint for the future of NHS and care services London: NHS England; 2016.
45. Noble AJ, Goldstein LH, Seed P, Glucksman E, Ridsdale L. Characteristics of people with epilepsy who attend emergency departments: Prospective study of metropolitan hospital attendees. Epilepsia. 2012;53(10):1820-8.
46. Allard J, Shankar R, Henley W, Brown A, McLean B, Jadav M, et al. Frequency and factors associated with emergency department attendance for people with epilepsy in a rural UK population. Epilepsy Behav. 2017;68(Supplement C):192-5.
47. Seddon N. Quite like heaven? Options for the NHS in a consumer age. London: Civitas; 2007.
48. Department of Health. The NHS Improvement Plan- Putting People at the Heart of Public Services. London: The Stationery Office; 2004.
49. Williams J, Petrov G, Kennedy U, Halpenny J, Doherty CP. Moving evidence based guidelines for seizures into practice in the emergency department: What's stopping us? Epilepsy Behav. 2017;72:72-7.
50. Department of Health. The NHS Constitution. London: Williams Lea; 2015.

For peer review only

For peer review only - http://bmjopen.bmj.com/site/about/guidelines.xhtml

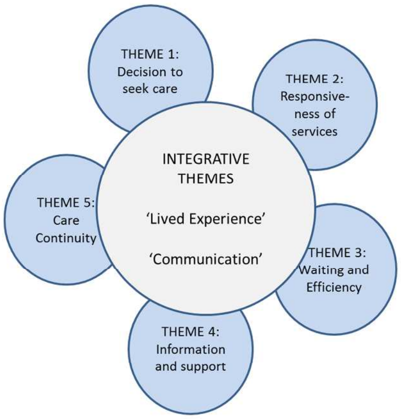

Fig.1 Schematic diagram demonstrating the five main themes identified in the study, surrounding the two central and integrative themes of 'lived experience' and 'communication'.

338x190mm (300 x 300 DPI)

SRQR Checklist

No.	Topic	Item	
	**Title and abstract**		
S1	Title	Concise description of the nature and topic of the study Identifying the study as qualitative or indicating the approach (e.g., ethnography, grounded theory) or data collection methods (e.g., interview, focus group) is recommended	S1- Page 2
S2	Abstract	Summary of key elements of the study using the abstract format of the intended publication; typically includes background, purpose, methods, results, and conclusions	S2- Page 2
	**Introduction**		
S3	Problem formulation	Description and significance of the problem/phenomenon studied; review of relevant theory and empirical work; problem statement	S3- Pages 3/4
S4	Purpose or research question	Purpose of the study and specific objectives or questions	S4- Page 6
	**Methods**		
S5	Qualitative approach and research paradigm	Qualitative approach (e.g., ethnography, grounded theory, case study, phenomenology, narrative research) and guiding theory if appropriate; identifying the research paradigm (e.g., postpositivist, constructivist/interpretivist) is also recommended; rationale[b]	S5- Page 2
S6	Researcher characteristics and reflexivity	Researchers' characteristics that may influence the research, including personal attributes, qualifications/experience, relationship with participants, assumptions, and/or presuppositions; potential or actual interaction between researchers' characteristics and the research questions, approach, methods, results, and/or transferability	S6- Page 6
S7	Context	Setting/site and salient contextual factors; rationale[b]	S7-Page5
S8	Sampling strategy	How and why research participants, documents, or events were selected; criteria for deciding when no further sampling was necessary (e.g., sampling saturation); rationale[b]	S8- Pages 8/9
S9	Ethical issues pertaining to human subjects	Documentation of approval by an appropriate ethics review board and participant consent, or explanation for lack thereof; other confidentiality and data security issues	S9- Page 7
S10	Data collection methods	Types of data collected; details of data collection procedures including (as appropriate) start and stop dates of data collection and analysis, iterative process, triangulation of sources/methods, and modification of procedures in response to evolving study findings; rationale[b]	S10- Pages 6, 8/9
S11	Data collection instruments and technologies	Description of instruments (e.g., interview guides, questionnaires) and devices (e.g., audio recorders) used for data collection; if/how the instrument(s) changed over the course of the study	S11- Page 7
S12	Units of study	Number and relevant characteristics of participants, documents, or events included in the study; level of participation (could be reported in results)	S12- Pages 8/9
S13	Data processing	Methods for processing data prior to and during analysis, including transcription, data entry, data management and security, verification of data integrity, data coding, and anonymization/deidentification of excerpts	S13- Pages 7/8
S14	Data analysis	Process by which inferences, themes, etc., were identified and developed, including the researchers involved in data analysis; usually references a specific paradigm or approach; rationale[b]	S14- Page 8
S15	Techniques to enhance trustworthiness	Techniques to enhance trustworthiness and credibility of data analysis (e.g., member checking, audit trail, triangulation); rationale[b]	S15- Page 8
	**Results/findings**		
S16	Synthesis and interpretation	Main findings (e.g., interpretations, inferences, and themes); might include development of a theory or model, or integration with prior research or theory	S16- Pages 10-18
S17	Links to empirical data	Evidence (e.g., quotes, field notes, text excerpts, photographs) to substantiate analytic findings	S17- Pages 10-18
	**Discussion**		
S18	Integration with prior work, implications, transferability, and contribution(s) to the field	Short summary of main findings; explanation of how findings and conclusions connect to, support, elaborate on, or challenge conclusions of earlier scholarship; discussion of scope of application/generalizability; identification of unique contribution(s) to scholarship in a discipline or field	S18- Pages 18-21
S19	Limitations	Trustworthiness and limitations of findings	S19- Pages 21/22
	**Other**		
S20	Conflicts of interest	Potential sources of influence or perceived influence on study conduct and conclusions; how these were managed	S20- Page 23
S21	Funding	Sources of funding and other support; role of funders in data collection, interpretation, and reporting	S21- Page23

[a] The authors created the SRQR by searching the literature to identify guidelines, reporting standards, and critical appraisal criteria for qualitative research; reviewing the reference lists of retrieved sources; and contacting experts to gain feedback. The SRQR aims to improve the transparency of all aspects of qualitative research by providing clear standards for reporting qualitative research.

[b] The rationale should briefly discuss the justification for choosing that theory, approach, method, or technique rather than other options available, the assumptions and limitations implicit in those choices, and how those choices influence study conclusions and transferability. As appropriate, the rationale for several items might be discussed together.

ACADEMIC MEDICINE
